# Hybrid Materials Based on Fly Ash, Metakaolin, and Cement for 3D Printing

**DOI:** 10.3390/ma14226874

**Published:** 2021-11-15

**Authors:** Joanna Marczyk, Celina Ziejewska, Szymon Gądek, Kinga Korniejenko, Michał Łach, Mateusz Góra, Izabela Kurek, Neslihan Doğan-Sağlamtimur, Marek Hebda, Magdalena Szechyńska-Hebda

**Affiliations:** 1 Faculty of Material Engineering and Physics, Cracow University of Technology, Warszawska 24, 31-155 Kraków, Poland; joanna.marczyk@pk.edu.pl (J.M.); celina.ziejewska@pk.edu.pl (C.Z.); szymon.gadek@pk.edu.pl (S.G.); kinga.korniejenko@pk.edu.pl (K.K.); michal.lach@pk.edu.pl (M.Ł.); mateusz.gora@atmat.pl (M.G.); izabelakurek16@gmail.com (I.K.); 2 ATMAT Sp. z o.o., Siwka 17, 31-588 Kraków, Poland; 3 Department of Environmental Engineering, Faculty of Engineering, Nigde Omer Halisdemir University, 51240 Nigde, Turkey; neslihandogansaglamtimur@gmail.com; 4 Plant Breeding and Acclimatization Institute—National Research Institute, Radzików, 05-870 Błonie, Poland

**Keywords:** 3D printing, hybrids, fly ash, concrete, metakaolin

## Abstract

Nowadays, one very dynamic development of 3D printing technology is required in the construction industry. However, the full implementation of this technology requires the optimization of the entire process, starting from the design of printing ideas, and ending with the development and implementation of new materials. The article presents, for the first time, the development of hybrid materials based on a geopolymer or ordinary Portland cement matrix that can be used for various 3D concrete-printing methods. Raw materials used in the research were defined by particle size distribution, specific surface area, morphology by scanning electron microscopy, X-ray diffraction, thermal analysis, radioactivity tests, X-ray fluorescence, Fourier transform infrared spectroscopy and leaching. The geopolymers, concrete, and hybrid samples were described according to compressive strength, flexural strength, and abrasion resistance. The study also evaluates the influence of the liquid-to-solid ratio on the properties of geopolymers, based on fly ash (FA) and metakaolin (MK). Printing tests of the analyzed mixtures were also carried out and their suitability for various applications related to 3D printing technology was assessed. Geopolymers and hybrids based on a geopolymer matrix with the addition of 5% cement resulted in the final materials behaving similarly to a non-Newtonian fluid. Without additional treatments, this type of material can be successfully used to fill the molds. The hybrid materials based on cement with a 5% addition of geopolymer, based on both FA and MK, enabled precise detail printing.

## 1. Introduction

During the last several decades, the impact of Portland cement on the environment has been the subject of discussion for many researchers [1,2]. Portland cement is a traditional and indispensable material widely used in construction around the world [3,4]. Due to its main advantages, including resistance to fire, rust, and rot, as well as flexibility in molding and shaping, cement is one of the most frequently used materials in the construction industry [5,6,7]. However, cement production has led to negative environmental impacts. The process consumes a large number of raw materials and has high energy requirements, produces a large amount of carbon dioxide that is released into the atmosphere, and thus contributes to global warming [4,5,6,8]. This disadvantage leads to the need for the development of new alternative materials and methods, including geopolymer binders.

The term ‘geopolymer’ was first used by Joseph Davidovits in the 1970s [3,9]. It defines the class of inorganic polymers, usually received by mixing metakaolin, fly ash, or slag with an alkaline activator [5,10,11] and containing the Al and Si tetrahedron network [12]. Metakaolin (MK) is a de-hydroxylated form of clay kaolin mineral [6,7,8,9,10,11,12,13,14], preferred in geopolymer production due to its bright color, easy control of the Si/Al ratio, and effectiveness of geopolymerization reactions; however, MK is relatively expensive [15]. Fly ash (FA), a by-product of coal power plants, is also a frequently used source material; however, its chemical composition is difficult to control, and the quality of FA depends on the type of coal and power-plant efficiency [16]. Commonly used alkaline activators are sodium hydroxide (NaOH), potassium hydroxide (KOH), or their combination, together with sodium silicate or potassium silicate [15,17,18]. The geopolymerization process occurs in three steps: (i) dissolution of the aluminosilicate material in an activator solution; (ii) transportation or diffusion of Al and Si ions and the formation of small, coagulated structures; and (iii) their polycondensation to form hydrated products [19,20]. Many natural minerals [21,22,23], calcined clays [24,25,26], and industrial by-products such as blast furnace slag [27], fly ash [28,29,30,31,32], rice husk ash [33], waste glass [34], and red mud [35] can be used as additional materials for the synthesis of geopolymers [17,36]. Therefore, geopolymer technology is an important solution for industrial waste utilization, the amount of which increases every year [8,17]. The reuse of by-product materials contributes to the reduction of greenhouse gas emissions and is an energy-saving and ecological alternative to Ordinary Portland Cement (OPC) [37,38,39,40].

The geopolymers and Portland cement have comparable properties [5,41,42]. They are characterized by high compressive strength [43], fire resistance [44], incombustibility, good resistance to chemical attack [45], high fracture toughness [46], low shrinkage [47], low thermal conductivity [44], low permeability [16], high stability at elevated temperatures [13], high strength-to-weight ratio, good durability and strong bonding, excellent heavy metal immobilization [12], resistance to freeze-thaw cycles [48], and low manufacturing energy consumption for construction purposes [49]. Their mechanical properties can be controlled in a wide range. Geopolymers have been successfully applied in a number of structural construction applications, such as beams, columns, slabs, tunnel linings, paving, etc.

A geopolymer hybrid is a combination of geopolymer and OPC or other binders, in order to produce material that combines the positive properties of OPC with the properties of alkali-activated materials or geopolymers. Hybrid OPC-geopolymer concrete has a lowered carbon footprint and improved ambient temperature curing while maintaining the positive properties of heavy metals immobilization, generally good mechanical properties, and high durability [50,51]. In the case of hybrid OPC-geopolymer concrete, generally of high FA content (70–90%) and low OPC content (10–30%), the clinker reaction products and reaction products from the glass phases of FA coexist. As the curing time progresses, when in combination, the (N,C)-A-S-H-type gels, called “hybrid gels”, and aluminum-modified calcium silicate hydrate C-A-S-H-type gels densify the cementitious matrix and are responsible for the mechanical performance of this type of material [52].

In the construction industry, 3D printing is considered environmentally friendly, due to its offering designing freedom, automation, less waste generation, reduced raw material consumption, and lower labor cost [53]. Significant progress has been made toward the construction of 3D concrete printers (3DCP); the printing methods are focused mainly on the pumping and extrusion of cementitious pastes to generate buildable layers. An extrusion-based three-dimensional concrete printing (E3DCP) process has still not been widely applied, mainly due to technical hurdles related to materials development and processing challenges. The current, severely limited, scope of materials that can be used in the 3D printer includes rapid-hardening Portland cement (RHPC), calcium aluminate cement (CAC), magnesium oxychloride cement, fiber-reinforced cement polymer, and ultra-high-performance concrete (UHPC). It is urgently necessary to develop mixes that can be sufficiently fluid and at the same time have sufficient viscosity to retain their shape after the printing process. The printed layers must be self-supporting and free of those discontinuity flaws caused by unwarranted stiffness and insufficient cohesion. In E3DCP, shrinkage due to drying or post-processing is another limitation. Altogether, this results in limited 3DCP applications. These methods still represent only 3% of the total additive manufacturing industry [54,55]. Supplementary cementitious materials (e.g., metakaolin or fly ash) are frequently utilized as a fractional replacement for cement to augment the desired rheological and packing properties. Geopolymers have also been recognized as a promising construction material for the 3D printing process due to their fast setting, the wide range of optimizable parameters, and their advantages in combination with substances modifying these properties [56].

None of the literature informs about the results of research on products made from concrete-geopolymer hybrids and their use in the 3D printing process. Therefore, we address in this article the development of materials based on hybrid geopolymers and OPC that can be used in the different methods of 3DCP.

## 2. Materials and Methods

### 2.1. Raw Materials

The commercial cement CEM I 42.5R from the cement plant Górażdże Cement S.A. (Heidelberg Cement Group, Chorula, Poland) was used for the experiments. According to manufacturer protocol, in appropriate proportions, after very fine grinding and homogenization, the raw material was heated (cyclone heat exchangers) and then sintered (rotary furnace; raw material temperature 1450 °C, flame and gas temperatures 2000 °C). The material remained in the high-temperature zone for approx. 30 min. The temperature of cement clinker at the exit of the furnace was approx. 900–1300 °C. Then it was subjected to intensive cooling, down to a temperature of about 100 °C. As a result, the cement clinker (in the form of hard sintered lumps) was obtained. The product, with the addition of gypsum, was ground in a ball mill to a very fine powder (CEM I Portland cement). This cement meets the standard requirements according to PN-EN 197-1 [57], and the properties described in the Declaration of Performance No. 1487-CPR-027-02. Cement conforms to the IBDiM Technical Recommendation No. RT/2010-02-0060/1.

The fly ash (FA) from the combined heat and power plant in Skawina (Skawina CHP Coal Power Plant, Skawina, Poland) and metakaolin (MK) KM 60 (Keramost, Kadaň, Czech Republic) were used as raw materials for geopolymers production. The pulverization process of FA was used to uniform the chemical composition and particle size, as FA was collected from different mechanical and electrostatic precipitators and zones. MK was prepared via the dehydroxylation of kaolin to remove the chemically bonded hydroxyl ions, according to the procedure described earlier [58,59,60]. The raw materials were mixed with commercial quartz sand with a chemical composition: 90.0–90.3% SiO_2_, max. 0.2% Fe_2_O_3_, 0.08–0.1% TiO_2_, 0.4–0.7% Al_2_O_3_, 0.17% CaO, 0.01% MgO.

### 2.2. Characterization of Raw Materials

The chemical composition of starting materials was analyzed using X-ray fluorescence (PANalytical Epsilon 3 XLE, Malvern Panalytical, Lelyweg 1, Almelo, The Netherlands) according to PN-EN 1744-1+A1:2013 [61].

The mineralogical characterization was carried out using X-ray diffraction (XRD) PANalytical Aeris (Malvern Panalytical, Lelyweg 1, Almelo, The Netherlands), using CuKα radiation. Samples were scanned in the angular range from 10° to 70° (2θ) at 0.003° (2θ) step size and a time per step of 340 s. The qualitative and quantitative analysis was performed against the ICDD (International Center for Diffraction Data, PDF-4) catalog and the HighScore Plus software (PANalytical).

The structural properties were determined using a Fourier transform infrared spectroscopy (FTIR) spectrometer (Shimadzu IRAffinity-1S, Kioto, Japan) equipped with the ATR Quest (Specec) adapter. The wavenumber range was 4000 cm^−1^ to 400 cm^−1^. A total of 32 spectra was averaged to reduce noise levels. The spectra were analyzed using a database with Shimadzu LabSolution FTIR software.

Water content was determined by the weight method in accordance with the standard, PN-EN 15934:2013 [62].

The pH value was determined using the potentiometric method (2.0–12.0 measuring range with 0.2 uncertainty) in accordance with the standard, PN-EN ISO 10523:2012 [63].

Water-leaching tests were carried out in accordance with PN-EN 12457-2:2006 [64]. In the aqueous extracts, the pH was also determined.

The concentrations of natural radioactivity (^40^K, ^226^Ra, ^228^Th) in the raw materials were tested using the high-resolution gamma spectrometry method. A device was equipped with a high-purity germanium detector (HPGe). In accordance with the regulation of the Council of Ministers, in the case of “requirements regarding the content of natural radioactive isotopes in raw materials and materials used in buildings for human and livestock habitation, as well as in industrial waste used in construction, and the control of the content of these isotopes”, building materials are qualified on the basis of two activity indicators, defined according to the following relationships [65]:

(1) $$\begin{array}{l} f_{1}=\frac{C_{K}}{3000\mathrm{Bq}/\mathrm{kg}}+\frac{C_{Ra}}{300\mathrm{Bq}/\mathrm{kg}}+\frac{C_{Th}}{200\mathrm{Bq}/\mathrm{kg}}\\ f_{2}=C_{Ra} \end{array}$$

where C*_K_*, C*_Ra_*, and C*_Th_* are the isotope concentrations of potassium ^40^K, radium ^226^Ra and thorium ^228^Th, expressed in Bq kg^−1^.

The morphology of the raw material particles was observed via a scanning electron microscope (JEOL JSM5510LV, JEOL, Tokyo, Japan). Particles of fly ash and metakaolin were stuck onto carbon tape to ensure good conductivity. The samples were coated with a layer of gold, using a vacuum evaporator (BS300).

The particle size distribution of fly ash and metakaolin was carried out using a laser particle size analyzer (FRITSCH ANALYSETTE 22 MicroTec plus, Fritsch GmBH, Idar-Oberstein, Germany). The volume-size distribution was expressed as D_10_, D_50_ (median), and D_90_.

The true density of the raw materials was determined using a helium pycnometer (Pycnomatic ATC, Thermo Fisher Scientific, Waltham, MA, United States).

The specific surface area was determined as a function of relative pressure with the BET (Brunauer–Emmett–Teller) method, using a physical sorption analyzer, Quantachrome Autosorb iQ—MP (Anton Paar company, Graz, Austria). The pore volume and the average pore size were determined by nitrogen adsorption/desorption using the BJH (Barrett–Joyner–Halenda) technique. The sample degassing temperature was 300 °C, the rate 20 °C min^−1^, and the soak time 180 min. Volume measurements of nitrogen adsorption and desorption were carried out at relative pressures (p/p_0_) in the range from 0.021 to 0.994 for 44 measuring points. The results were analyzed using the ASiQwin software.

Differential thermal analysis (DTA), coupled with thermogravimetry (TG, DTG) and evolved gas analysis (QMS), was performed with NETZSCH STA 449F3 and quadrupole mass spectrometry QMS 403 (Netzsch GmBH, Selb, Germany). The experiments were carried out in the temperature range from 30 °C to 1000 °C. Samples were heated at 10 °C min^−1^ in an air atmosphere. The data were analyzed using Proteus software (Netzsch). The TG curve is the change in mass loss and the DTA curve is the mass loss rate as a function of temperature [66,67].

### 2.3. Preparation of Geopolymer Specimens

FA or MK was mixed with sand in a 1:1 proportion and then activated. The activator solution consisted of 10 M sodium hydroxide and the aqueous solution of sodium silicate (R-145, a molar ratio of 2.5 and density of 1.45 g cm^−3^). The ratio of sodium hydroxide solution to sodium water glass was fixed at 1:2.5. Ingredient-mixing for 15 min in a low-speed mixer was performed 24 h before use, to allow the equilibration of a constant concentration and temperature. Six geopolymer types were designed, depending on composition and the liquid-to-solid ratio (Table 1).

The ingredients were mixed in a GEOLAB cement mortar mixer (Geolab, Warsaw, Poland) for 15 min to a homogeneous paste. The fresh geopolymer pastes were formed in the molds with the size of 50 mm × 50 mm × 50 mm for compressive strength test; 71 mm × 71 mm × 71 mm for the abrasion resistance test; 200 mm × 50 mm × 50 mm for the flexural strength test. Molds were shaken to remove the trapped air. The specimens were cured at 24 h at 75 °C, and then de-molded and stored at ambient conditions.

### 2.4. Characterization of Geopolymers

The compressive strength of the geopolymers after curing for 1 and 28 days was tested in accordance with the PN-EN 12390-3:2019 standard [68] on a testing machine, a MATEST 3000 kN (Model C-104 with Cyber-plus evolution program, MATEST S.p.A., Arcore, Italy).

A flexural strength test of geopolymer samples after curing for 28 days was acquired in accordance with the PN-EN 12390-5:2019 standard with a concrete press (MATEST).

The surface abrasion resistance test was conducted in accordance with the PN-EN 13892-3:2015 standard [69] using the Böhme abrasion test abrader. The samples were weighed, placed on the steel test disc, and 20 g of abrasive powder (artificial corundum) was spread over the grinding path. During the test, the sample was subjected to 16 abrasion cycles consisting of 22 revolutions. After every 22 revolutions, the abrasive powder was replaced with fresh powder. The specimen was turned about the vertical axis by 90° after each cycle. The samples were weighed after the experiment was completed. The average weight loss and volume decrease were calculated as follows:

(2) $$\mathrm{Surface}\;\mathrm{abrasion}\;\mathrm{weight}\;\mathrm{loss},\;\%=\frac{w_{1}-w_{2}}{w_{1}}\times 100$$

where *w*_1_ was the initial weight of the sample; *w*_2_ was the final weight of the sample [5].

Volumetric abrasion losses (cm^3^ 50 cm^−2^), ΔV = Δm/ρ (3)

where Δm was the weight loss after 16 cycles; ρ was the density in g cm^−3^ [70].

The fire resistance test of the geopolymers was acquired in accordance with the PN-EN ISO 1182:2020 standard [71] in an electric furnace. The samples were dried at 60 °C and then cooled to ambient temperature. The test was carried out for 30 min at 750 °C. The weight of the specimens was measured before and after the experiment. The weight loss was expressed as a percentage of the initial sample weight [72]:

(4) $$Loss\;of\;mass,\;\%=100\times \left[1-\frac{mass\;after\;experiment}{mass\;before\;experiment}\right].$$

### 2.5. Hybrid Preparation

Geopolymer-based hybrids, made of both fly ash and metakaolin, were prepared from the CEM I 42.5R cement solution and the geopolymer mass. CEM I 42.5R cement solution was prepared in the amount of 5 wt %. Geopolymer mixtures made on the basis of fly ash contained an additive of sand (1:1) and the liquid-to-solid ratio was 0.28. Geopolymers made with a base of metakaolin contained an additive of sand (1:1), and the liquid-to-solid ratio was 0.35. Concrete control (without geopolymers) was made with a base of cement and sand in the proportion of 1:1, and the water-to-solid ratio was 0.125.

The GALAXY printer supplied by the ATMAT company (ATMAT, Kraków, Poland) was used for the printing process. About 50 kg of each tested material was prepared for printing. The printing process was carried out at an ambient temperature, with a printing speed of 150 mm s^−1^. The diameter of the nozzle was 15 mm, and the thickness of the applied layers was 10 mm.

### 2.6. Statistical Analysis

All data are the average of three to six repetitions. The standard deviation was calculated and is presented.

## 3. Results and Discussion

### 3.1. Characterization of Raw Materials

CEM I 42.5R was selected for the tests. It has a composition compliant with the requirements of PN-EN 197-1 [57]. Dry Portland cement is a white/grey, odorless, fine-ground material. The particle size was 5–30 µm. The specific density was 3.11 g cm^−3^, and the bulk density 1.42 t m^−3^. The main component was cement clinker (95%: alit, 3CaO•SiO_2_, ref. code: 00-016-0407; belit, 2CaO•SiO_2_, ref. code: 00-033-0303; tricalcium aluminate 3CaO•Al_2_O_3_, ref. code: 00-038-1429; brownmillerite 4CaO•Al_2_O_3_•Fe_2_O_3_, ref. code: 00-011-0124) mixed with gypsum (max. 5%, CaSO_4_•2H_2_O, ref. code: 00-006-0047), which was used as a setting-time regulator. The sulfate content (as SO_3_) reached the value of 3.24% (with the norm not exceeded, 4.00%), the chloride content (as Cl^−^), 0.06% (max. 0.100%), the alkali content (as Na_2_Oeq), 0.75%. The content of soluble chromium (VI) in the cement, due to its natural composition, was below 0.0002% of the total dry weight. The content of individual clinker phases has a significant impact on the course of the hydration process, an exothermic reaction of cement (clinker) with water. The amount of the total heat effect is determined by the presence of alite and tricalcium aluminate. On the other hand, alite and belite are the phases responsible for the buildup of strength. Therefore, in the early period of hydration, the presence of alite is of key importance for strength, while the content of belite over a longer period of time determines the strength. The pH of the studied cement was 11.0–13.5 at a temperature of 20 °C in water for a water–material ratio of 1:2. The melting-point value for cement was >1250 °C.

**Figure 1:**
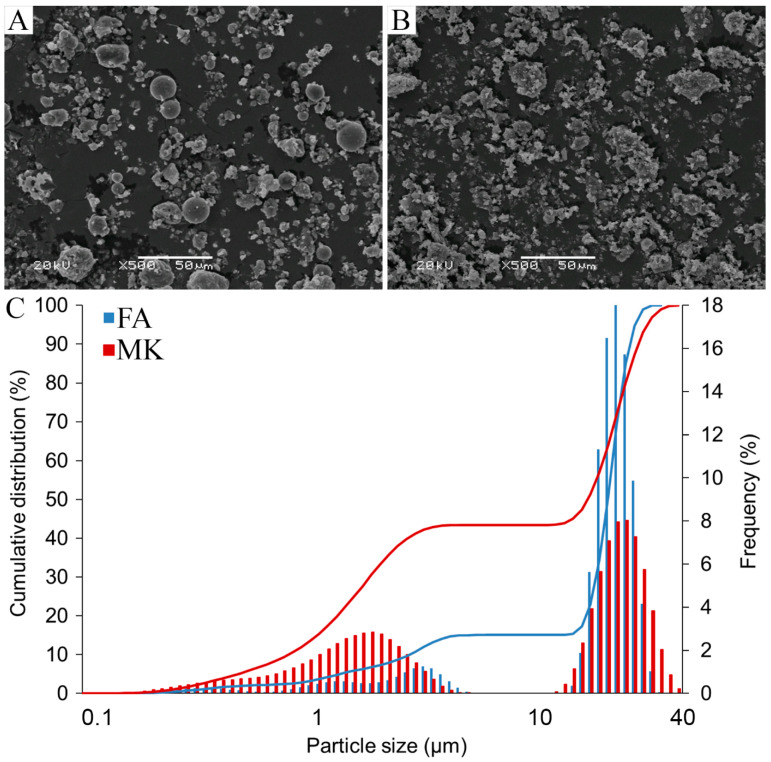
SEM micrographs (**A**,**B**) and particle size distribution (**C**) of fly ash and metakaolin. (**A**)—fly ash morphology, (**B**)—metakaolin morphology, (**C**)—particle size distribution described by cumulative distribution (lines) and frequency (bars).

Fly ash (FA) and metakaolin (MK) were considered as important raw materials for geopolymer production; however, both FA and MK’s suitability for geopolymerization reactions depended on their physical properties. The particles of FA had approximately spherical morphology, which is beneficial in order to achieve a successful geopolymerization process (Figure 1A and Figure S1A,C,E in Supplementary Materials). It improves the rheological properties of the mixture, increasing its workability. In addition, it reduces the need for liquid substances and has a beneficial effect on the mechanical properties of geopolymers [73]. In contrast, the particles of MK were in the form of irregular flakes with random geometry, rough and porous surface texture, and tend to form agglomerates (Figure 1B and Figure S1B,D,F in Supplementary Materials). The morphology of both materials seems to be typical, as described earlier [74]. FA and MK also had different particle-size distributions (Figure 1C, Table S1 in Supplementary Materials). The FA particle size ranged from 1.3 to 32.5 μm, with 90% particles of less than 30 µm and a distribution width of D_50_ 22.3 μm; the MK particle size ranged from 0.5 to 39.2 μm, with 90% of the particles’ size exceeding 30 µm, and a distribution width of D_50_ 18 μm. Along with a decrease in the particle size, the density and mechanical properties of the geopolymer have increased. The phenomenon is attributable mainly to the greater surface area available for chemical reactions. The smaller particles have a larger surface area in comparison to the volume and, thus, higher reactivity, including the rate of dissolution of the monomers, i.e., silicate and aluminate, consequently showing a more effective geopolymerization process [75,76,77,78]. Furthermore, the porosity is the lowest at the smallest particle size and the voids can be better filled within fine particles, leading to denser and stronger geopolymer products [79]. In earlier studies, we showed the beneficial effect of grinding the raw materials, even if the process is energy-intensive; the reduction of particle size and the increase of specific surface area were crucial to obtaining the higher reaction rate of the precursor, more reacted final material and the proper mechanical properties of the geopolymer products [80]. The size reduction throughout the milling process is recognized as a mechanical activation of the material, resulting in an increase in compressive strength [75,79]. Another way is separating in different fractions to enable smaller particles [81]. On the other hand, if gases can be released from the raw materials through the voids between particles in a larger fraction, they do not affect the mechanical strength as much as when they destroy the compactness of the material, leaving it with lower density and lower compressive and bending strength.

The density of the FA was 2.288 ± 0.001 g cm^−3^, whereas the MK density of 2.566 ± 0.001 g cm^−3^ was compliant with the specifications presented by the manufacturer (2.50–2.80 g cm^−3^) and other authors [82,83].

To yield information concerning the effects of surface porosity and particle size for the FA and MK, external area and pore area evaluations were conducted (Table 2 and Table S1 in Supplementary Materials, Figure S2 in Supplementary Materials). Depending on the method, the value of the surface area for FA was in the range of 10.431–14.616 m^2^ g^−1^, and for MK the range was 12.999–21.415 m^2^ g^−1^, while the pore volume was 0.026–0.027 cm^3^ g^−1^ and 0.140–0.142 cm^3^ g^−1^ for FA and MK, respectively (Table 2, Figure S2 in Supplementary Materials). Similarly, the pore size was 2.134 nm for FA and 2.975 nm for MK. Therefore, the materials were defined as mesoporous, according to IUPAC classification [84,85,86,87], with slit-like interparticle pores [88,89,90] (Figure S1E,F in Supplementary Materials).

Fly ash and metakaolin are considered important raw materials for geopolymer production, as SiO_2_ and Al_2_O_3_ are their main chemical constituents (Table 3). The analysis of the chemical composition of FA and MK showed a high content of silica and alumina, which exceeded 70% and 90% in FA and MK, respectively. However, considering the Si:Al ratio, it is important to note that the raw materials before geopolymerization had Si:Al ratios of 3.26 and 2.17 for FA and MK samples, respectively. The mechanical properties of the geopolymers became increasingly elastic with the increasing total SiO_2_ content in the raw materials. On the other hand, homogeneity of the microstructure meant a compressive strength increase along with the Si:Al ratio [59]. Thus, although the total Si content can improve the properties of MK-originated geopolymers rather than FA-originated geopolymers, the final effect may be the complementary result of both factors—not only the total content of the Si and Al components but also their relationship to each other. The element differentiating significantly between both raw materials was calcium content; FA had a higher calcium content (5.120%, classified as class F), while a much lower content was found in MK (0.490%). The effect of calcium on geopolymer produced from metakaolin and fly ash is usually positive; a composite system with geopolymer gel and calcium–silicate–hydrate gel can co-exist when the calcium content increases [91]. The presence of CaO, together with MgO, which was much higher in FA than MK (Table 3), can contribute to increased pH value in the range of 10.0–13.0 [92]. Indeed, the leachate for raw materials was highly alkaline (pH value > 12.0) for FA, and slightly acidic (pH value = 6.0) for MK at a temperature of 20 °C; the high pH value was primarily due to the hydrolysis of lime, which yields free hydroxyl ions. Furthermore, numerous accessory minerals of Ba, Sr, Zn, Pb, V, Cr, Cu, Ni, Rb, Ga, Zr, Te, As, Sb, Sn, and Y were typical components of FA, while they were present to a lesser extent in MK (Table 3). Accordingly, the more rich and complex composition of FA resulted in more abundant total dissolved substances in the FA leachate when compared to that of MK (Supplementary Materials Table S2). Generally, FA is a hazardous material collected from coal production as an unburned residual; thus, its potentially detrimental role in polluting the environment should be carefully recognized. However, the detailed chemical analysis of leachate from raw materials revealed the presence of toxic elements at an acceptable level, in the case of both FA and MK (according to EU Decision 2003/33/EC [93], and Chinese National Standard GB 5085.3-2007 [94]), even if a much higher content of Sb, Ba, Cr, Cr(VI), Mo, Hg, Se, chlorides, fluorides, and sulfates were detected in FA (Supplementary Materials Table S2). Similarly, the ^40^K, ^226^Ra, and ^228^Th radioactivity of FA and MK were below the international limits (EU report [95]); they slightly exceeded an average radioactivity level for FA, but not for MK, as produced in Europe (622–793 Bq kg^−1^ for ^40^K; 126–191 Bq kg^−1^ for ^226^Ra; and 89–91 Bq kg^−1^ for ^232^Th) [96] (Supplementary Materials Table S3).

A greater value for loss on ignition (LOI), an indicator of the residual carbon content [97], was observed in the case of FA (3.284%) than in MK (0.722%) (Table 3) and, similarly, the dissolved organic carbon (DOC) level was higher in FA leachates (Supplementary Materials Table S2). Many countries have recently tended to institute more strict specifications for the limit on LOI (ranging from 3% to 6%) for quality assurance. Although obtained LOI values are common (e.g., [98,99]), it is worth noticing that the residual carbon present in fly ash can absorb water and chemical admixtures (e.g., superplasticizer, air-entraining agent), reducing their efficiency or even resulting in a changed air–void system in the concrete. The LOI results were confirmed by higher FA instability and organic decomposition, along with a temperature rise during thermal analysis (Figure S3 in Supplementary Materials). Although the FA and MK samples showed low weight loss (Figure S3A in Supplementary Materials) and related thermal effects (Figure S3B in Supplementary Materials) in the temperature range from 25 °C to 400 °C, the extreme differences between both materials were recorded at 400–700 °C, with the maximum at 583 °C (Figure S3A–H in Supplementary Materials). Significant mass loss and exothermal effects were recorded for FA, but they were not observed for MK. These resulted from organic material decomposition due to C (*m/z* 12, Figure S3C in the Supplementary Materials), and CO_2_ (*m/z* 44, Figure S3H in the Supplementary Materials) products were recorded with the QMS method. A similar pattern of changes in the temperature range of 400–700 °C was not found for H_2_O (*m/z* 17 and 18, Figure S2D,E in the Supplementary Materials), CO (*m/z* 28, Figure S2F in the Supplementary Materials), and O_2_ (*m/z* 32, Figure S2G in the Supplementary Materials). For H_2_O and O_2,_ only the evaporation effects were recorded in the temperature range from 25 °C to 125 °C, with higher values for FA. This can result from capillary effects, which occur due to the higher adhesive and cohesive forces interacting between the H_2_O (and O_2_) and the internal surface of pores in raw-material particles. This finding is confirmed by the FA microstructure, described above, i.e., smaller particle size, particles’ total pore volume, and average pore diameter, which altogether influence the total surface area (much smaller for FA). Such microstructures allow keeping the H_2_O (and O_2_) molecules more closely bonded to the FA pore surface than to bigger particles and the pores of MK at room temperature. When the temperature rises, adsorbed water (and dissolved O_2_) can be removed to the atmosphere to a greater extent. In contrast, the water is able to penetrate the MK easily through the larger number of capillary channels, and a higher water amount can be removed to the atmosphere at room temperature. This effect is also compatible with higher Ca content in FA. The greater bonding energy of the calcium, the reduction of the repulsive forces between the particles, the van der Waal’s forces, and the greater misfit of the calcium ion and its hydration shell would tend greatly to reduce the number of water layers that could be adsorbed.

The qualitative results of XRD (Figure S4 in the Supplementary Materials) and their quantitative analysis with the Rietveld method (Table 3), performed for FA, revealed the presence of phases rich in Si and Al, such as mullite (Al_6_Si_2_O_13_, ref. code: 00-015-0776) and quartz (SiO_2_, ref. code: 01-075-8320). Furthermore, hematite (Fe_2_O_3_, ref. code: 04-002-7501), magnetite (Fe_3_O_4_, ref. code: 04-022-0447), anhydrite (CaSO_4_, ref. code: 00-003-0163), and rutile (TiO_2_, ref. code: 04-015-7316) were recorded for FA in decreasing order of presence. In MK, a different phase composition was recorded, with the main phases, rich in Si and Al, consisting of illite (K, H_3_O) Al_2_Si_3_AlO_10_(OH)_2_, ref. code: 00-026-0911), kaolinite (Al_2_Si_2_O_5_(OH)_4_, ref. code: 00-058-2004), quartz, and mullite. The different forms of Si/Al in FA and MK suggest the various potentials for geopolymerization processes [86,87,88]. The mullite and quartz phases in FA may not dissolve readily in an alkaline solution and, as a result, can lower the geopolymerization’s effectiveness. On the other hand, the crystalline quartz phase, due to the aluminosilicate compounds, can improve the physical and mechanical properties of geopolymers [99]. The kaolinite in MK may demonstrate an incomplete calcination process, which is dependent on the temperature treatment [80,100,101]. The asymmetrical hump appearing clearly in the range of 20–30° (2θ) is commonly identified in MKs and indicates an amorphous phase related to aluminosilicate glass (Figure S4 in the Supplementary Materials) [85,102]. In the case of MK, the crystalline structure can be broken down to form an amorphous phase during calcination, at a temperature lower than that necessary to generate a liquid phase and produce glass on cooling. However, the illite in MK has significant K_2_O content and, in the case of geopolymerization of the reacting minerals (dissolution and polycondensation), it can have a significant effect on the increase in strength of the geopolymerization products [25,103]. Moreover, it is worth noticing that the mineral composition can result in lower adhesive and cohesive forces interacting between the H_2_O and the internal surface of pores in MK particles (as described above), because kaolinite and illite favor the more rapid water sorption/desorption.

**Figure 2:**
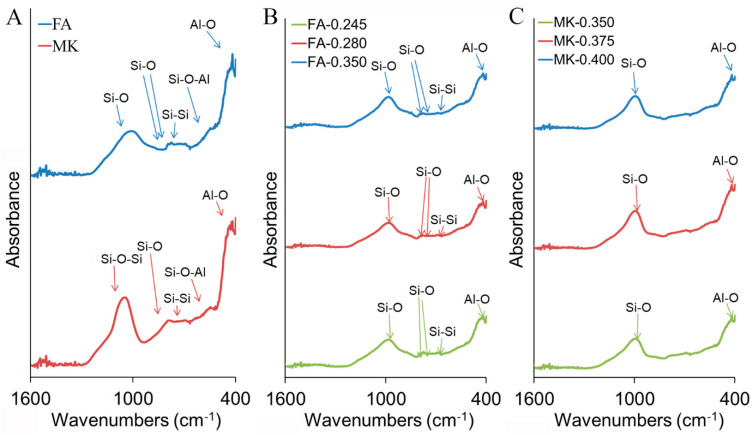
FTIR spectra of fly ash and metakaolin (**A**), as well as geopolymers produced from fly ash (**B**) and metakaolin (**C**), mixed with sand and NaOH: water glass in ratio 0.245, 0.280 and 0.350 for FA, and 0.350, 0.375, and 0.400 for MK. Complete spectra are presented in Figure S5 in the Supplementary Materials.

The results were confirmed by the FTIR spectrum (Figure 2, Table S4 in the Supplementary Materials). FTIR spectra contain information on the mineralogical composition as each mineral component has a unique absorption pattern in the mid-IR range and, thus, they are widely used for the study of aluminosilicates. The most intense band was recorded for both FA and MK at approximately 1000–1100 cm^−1^. FA was characterized by the maximum vibration at wavenumber 1003 cm^−1^, related to the asymmetric Si-O stretching vibrations occurring in the aluminosilicate structures [73]. In MK, the main band, centered at 1055 cm^−1^, was associated with the Si-O-Si vibrations originating from silicate minerals present in the material [104]. The band intensity around 1100 cm^−1^, related to the asymmetrical stretching vibration peak for the Si-O bond, was higher for MK than FA. Altogether, this is in agreement with a higher total content of silica in MK (Table 3). Moreover, common peaks found at 793 cm^−1^, associated with Si-O bending vibrations, peak at 699 cm^−1^ with Si-Si vibrations, peak at 547 cm^−1^ with Si-O-Al vibrations, and peak at 418 cm^−1^ with Al-O vibrations; all were higher for MK and were thus related to the higher total content of Si and Al. Similar shifts in raw materials have been observed previously [38,85,88]. Considering the mineral phase content, FA was characterized by the maximum vibration at wavenumber 1003 cm^−1^ and the bending vibration at 793 cm^−1^ and 781 cm^−1^ that can be attributed to the presence of quartz [38,105]. The presence of mullite in FA was represented by the band around 557 cm^−1^ related to the substitution of Al for Si in the mullite structure, and furthermore by the shift of the main band around 1000 cm^−1^ (in comparison to MK bands) and thus the marking band at 915 cm^−1^ associated with the presence of aluminum in the octahedral position, characteristic of mullite. MK phases can be assigned by the main band centered at 1055 cm^−1^ for kaolinite and 537–529 cm^−1^, 1023–1027 cm^−1^, and 1066–1070 cm^−1^ for illite.

### 3.2. Properties of Concrete and Geopolymers

Different cement powders are used in industrial installations for the production of binding materials, e.g., ready-mixed concrete of classes C 16/20—C 40/50 and higher, SCC self-compacting mortars, and concrete for large- and small-sized prefabricates. It is used both in professional conditions and by individual users for indoor and outdoor construction. In our experiments, we used cement powder, which required 27.2% water to achieve standard consistency. The beginning of the setting time of concrete was 227 min (with the normative requirement ≥ 60 min). The compressive strength after 2 days was 28.4 MPa (with the normative requirement ≥ 20 MPa), while after 28 days, it was 60.8 MPa (with the normative requirement ≥ 42.5 MPa ≤ 62.5 MPa). The volume stability was 0.6 mm (with the requirement ≤ 10 mm). The specific surface of the concrete was 4187 cm^2^ g^−1^.

Due to their excellent properties, geopolymer products have been used, among others, as refractory materials [42,100], thermal insulation [106], construction [107], and 3D printing [108]. Among the diverse range of potential applications, geopolymers can also be used as technologically advanced composites in planes, ships, the nuclear power industry [109], biomaterials, or ecological utilization, replacing plastic [58,59,110]. In our experiments, the raw materials for geopolymers were mixed with commercial sand, the composition of which includes SiO_2_ 90.0–90.3%, Fe_2_O_3_ max. 0.2%, TiO_2_ 0.08–0.1%, Al_2_O_3_ 0.4–0.7%, CaO 0.17%, and MgO 0.01% (particle size: < 50 µm). The liquid-to-solid mass ratio was maintained at 0.245, 0.280 and 0.350 for FA, while at 0.350, 0.375, and 0.400 for MK (Table 1). Compared to concrete, the setting time at room temperature was longer for geopolymers, up to 405 min (initial setting time)—630 min (final setting time) for fly ash-based geopolymers, and up to 323 min (initial setting time)—522 min (final setting time) for metakaolin-based geopolymers (Table S5, Supplementary Materials). It is worth noting that the setting time could be adjusted in a wide range, along with the changes in temperature during the setting process, the duration of mixing of ingredients before setting process and, in a narrower range, along with the changes in the liquid to solid ratio and geopolymer composition resulting from different raw materials (FA and MK). In the first case, an increase from room temperature to the temperature of 75 °C can reduce the setting time by even one order of magnitude. Prolonging mixing from 15 min to 30 min can shorten the setting time twice. Moreover, changing the liquid-to-solid ratio from 0.245 to 0.350 for FA and from 0.350 to 0.400 for MK can extend the setting time by approximately 40% and 25%, respectively. In differently composed geopolymers, the range of changes was slightly greater for FA pastes mixed for 15 min and kept at 75 °C during the setting time measurement (27–46 min) than MK geopolymers under the same conditions (28–39 min). The results were confirmed by the consistency of fresh geopolymer mortars, determined by the flow table method and the Novikov cone method (Table S6, Supplementary Materials). The mortars consistency can be defined as dense-plastic for FA-0.245, FA-0.280, and MK-0.350; plastic for MK-0.375 and MK-0.400; and liquid for FA-0.350, suggesting that the liquid-to-solid ratio chosen for FA geopolymers influences its properties in more extreme ranges than the ratio chosen for MK geopolymers.

The FTIR spectra of geopolymers produced from fly ash and metakaolin, with a different liquid-to-solid ratio and curing for 28 days, generally showed a similar pattern to raw materials and to each other (independently of the liquid-to-solid ratio), although a lower intensity and number of bands were detected for geopolymers, particularly for MK-originated geopolymers (Figure 2, Figure S5 in the Supplementary Materials). The most intensive bands for geopolymers were those found at around 1000 cm^−1^, related to the vibration of Si-O(Si) (Table S4 in the Supplementary Materials). However, a significant difference from the raw materials was the shift of these bands from 1003 cm^−1^ to 989 cm^−1^ for FA-originated geopolymers and from 1055 cm^−1^ to 992 cm^−1^ for MK-originated geopolymers. This indicated the formation of new amorphous aluminosilicate gel phases during the geopolymerization process. The position at around 1000–1100 cm^−1^ is indicative of the silica structure and, thus, with increasing values of wavenumbers around 990, lower Si atoms at the tetrahedral position, relating to enriched Si-O at the tetrahedral position. At the same time, the raw material bands at 547 cm^−1^, representing the vibration of Si-O-Al, are reduced in the geopolymer material (Table S4 in the Supplementary Materials); similarly, the band absorbance at 418 cm^−1^ and adjacent bands, relating to the vibration of Al-O, is lowered (Figure S5 in the Supplementary Materials). To conclude, the formation of both FA- and MK-originated geopolymer material is favored in one direction to form a poly(silate-siloxo) (-Si-O-Al-O-Si-O-) structure, in which is the ratio Si:Al = 2; a poly(silate-disiloxo) (-Si-O-Al-O-Si-O-Si-O) structure, Si:Al = 3; or even additional sialate links, when Si:Al > 3; rather than in the direction to form poly(silate) (-Si-O-Al-O-), in which ratio Si:Al = 1. Positive ions (Na^+^, K^+^, Ca^2+^) must be present in such framework cavities to balance the negative charge of Al^3+^ in IV-fold coordination. Therefore, an FA-originated geopolymer structure was not surprising, as a much higher content of positive ions was available already in FA (raw material). In MK-originated geopolymers, the arrangement consisted of a more equal share of individual structures, due to the vibration around Si-Si 690 cm^−1^ and bending vibration Si-O around 793 cm^−1^ and 783 cm^−1^ being reduced; thus, the relative effect of the Al-O bonds increased. The effect correlated with the Si:Al ratio for FA versus MK raw materials, as the Si:Al ratio of 3.26 and 2.17 were calculated for FA and MK, respectively (before the geopolymerization process). Additionally, one can conclude that by calculating the Si:Al ratio of raw materials, the formation of the structure during the geopolymerization process can be predicted.

With the increase in the Si:Al ratio, geopolymers generally show higher mechanical properties due to the increased Si-O-Si bonds and residual silica as reinforcement. Therefore, the positive mechanical effect was expected due to the chemical arrangement of fly ash components (Table 3, Figures S4 and S5, Table S4 in the Supplementary Materials) and was confirmed by the structure of FA-originated geopolymers (Figure 1, Figure S6 in the Supplementary Materials). Indeed, analysis of the compressive strength, flexural strength, and abrasion resistance showed better mechanical properties of the FA geopolymers in comparison with the MK geopolymers, provided that the geopolymers were cured for 28 days (Table 4).

When compared to the results of the compressive strength test performed 1 day after the geopolymerization reaction, a different effect was observed, indicating the solidification process of the geopolymer as a chemical reaction, with the generation of new structures. Incomplete geopolymer formation after 24 h of curing at 20 °C is a common phenomenon, and the formation of aluminosilicate networks with the transition from hexa-coordinated Al(VI) to tetra-coordinated Al(IV) during the following days was identified [111]. Therefore, considering the changing parameters after the geopolymerization process, it is necessary to adjust the length of the curing period and specify them in the time-function parameters of geopolymer products. Although the liquid (activators) to solid (raw materials) mass ratio did not influence the geopolymer structure significantly (similar FT-IR spectra, Figure 2 and Figure S5 in the Supplementary Materials), it was a key factor affecting the compressive strength of geopolymers [9] (Table 4). Values ranged between 25.45–68.34 MPa for FA and MK geopolymer products cured for 1 day and 28 days. Along with the improved amount of liquids/a reduced amount of used raw materials (L/S increase from 0.245 to 0.350 for FA and from 0.350 to 0.400 for MK), the compressive strength decreased ~43% and 64%, respectively, for FA and MK, after the first day of curing (Table 4). Although the change of L/S ratio from 0.33 to 0.60 can reduce the final compressive strength, even up to 60% [9] in our studies, this effect was slightly counteracted in the geopolymers FA-0.28 and FA-0.35 with the passage of time since the compressive strength improvement was observed on the 28th day of curing (14% and 59%, respectively). Thus, one can conclude that the compressive strength of cured FA geopolymers did not depend on the L/S ratio. In contrast, the compressive strength of MK-originated geopolymers increased along with time only for MK-0.4 (39%), while for MK-0.350 and MK-0.375 it significantly decreased (32% and 44%, respectively). In this case, the differences remained significant between MK-0.350 and MK-0.400 (up to 34%), and it can be concluded that the L/S ratio is a factor of great importance for MK-originated geopolymers (Table 4). This is due to the excess of activator solution increasing the water quantity in the mix; thus, improper shrinkage along with time led to crack formation. This adverse effect of water on geopolymerization is reported elsewhere [16,39,102,112,113]. In conclusion, the L/S ratio should be optimized in each case when the composition of raw materials is changed. These statements are also reinforced by a comparison of FA and MK geopolymers as a product of reactions performed at the same L/S ratio (0.350). The compressive strength was lower for the FA geopolymer than the MK geopolymer and the experiments showed an FA geopolymer morphology with more of a cracking structure (Figure S6 in the Supplementary Materials).

The L/S ratio affected the flexural strength (bending strength) of the specimens in the same way due to more cracks, resulting in a more fragile structure of the samples. FA and MK had a flexural strength higher for FA-0.245 and FA-0.280 when compared to FA-0.350, MK-0.375, and MK-0.400, while a comparison between FA-0.350 and MK-0.350 showed the better mechanical properties of MK-0.350 (Table 4). Thus, it can be stated that the highest flexural strength is developed for a geopolymer with a lower L/S ratio, and FA required a lower L/S ratio than MK for a successful geopolymerization process. Although the flexural strength was analyzed after 28 days, it was suggested that the effect of the alkaline solution-to-binder ratio on the flexural strength of the geopolymer mortar can stabilize within 7 days [114], and an increasing alkaline solution-to-binder ratio has a negative effect on the flexural strength.

Generally, the mechanical properties tested during a surface abrasion resistance test also followed compressive and flexural strength changes (Table 4), and this is in agreement with the fact that the abrasion resistance increased along with the decrease in the ratio of L/S [115]. The only exception was MK-0.350, with the lowest parameter values.

### 3.3. Concrete and Geopolymer Hybrid Materials and Their 3D Printing

The above-described research was aimed at selecting the protocol and choosing the parameters allowing their implementation to produce optimal geopolymer products that could potentially be used for printing composite materials using the 3D printing system. The effect of the cement-to-geopolymer ratio on preparing the optimal consistency of mortar and thus defined setting time should be the main factor. However, the influence of both the duration of mixing time and the temperature of the mixture on the setting time could also be critical, considering the 3D printing conditions, because the material is mixed and heated as a result of friction immediately before feeding it to the printing nozzle. Therefore, the final print result depends not only on the fixed printer parameters but also on the mass sensitivity to mechanical and physical factors. A pilot test was designed to include both different cement-to-geopolymer ratios and parameters indirectly influencing the setting time. With the introduction of cement to geopolymer mortar, the setting time of the hybrid material was shortened (Figure S7, Supplementary Materials) as expected due to the differences in the setting time of both components (227 min for concrete and 630 min for geopolymer (Table S5, Supplementary Materials)). Surprisingly, this effect was sharp and linear only in the range of 5–30% cement in the hybrid material. Higher cement content (95% and 100%) prolonged the setting time in comparison to the results obtained for the range 5–30%. Further experiments were performed preferentially for materials based on FA-0.280 + 5% cement and MK-0.350 + 5% cement due to the best mechanical properties described earlier for geopolymers (Table 4). The general effect for both materials was similar, independently of the type of raw material used (Table S7, Supplementary Materials). However, the setting time for the hybrid materials based on MK-0.350 + 5% cement was shorter than the setting time for hybrid materials based on FA-0.280 + 5% cement, even if the liquid-to-solid ratio could suggest an opposite tendency. The differences in the setting time of hybrids were higher when the temperature of fresh mortars increased (Table S7, Supplementary Materials) and was related to differences in the setting time of geopolymers (MK vs. FA, mixed 15 min and measured at RT (Table S5, Supplementary Materials)). In both cases, the final result was determined by a few factors: (1) the higher porosity and specific surface area of MK than FA requires more water for the same workability of MK and FA materials (Figure 1, Table 2, Table S1 and Figure S2, Supplementary Materials); (2) the microstructure causes water to be more strongly absorbed in FA (van der Waals forces) while evaporating more easily at room temperature from MK (Figure S3, Supplementary Materials); (3) the values of the consistency of fresh geopolymers indicated that the FA geopolymer was more plastic than the MK geopolymer (Table S6, Supplementary Materials). Moreover, the results were consistent with the particle size distribution of the raw materials. The fluidity of paste with bigger and more round particles is lower [116], as observed for FA-originated material, in contrast to metakaolin’s smaller particles with a larger surface area and rough shape that may lead to interlocking between particles in the fresh paste. The interparticle forces are influential in regulating the rheology of the suspension at the high solid concentration. The net interparticle forces are governed by the sum of the attractive van der Waals and the electrostatic repulsive forces. With a change in particle size, both are altered and the interparticle forces with a finer particle are stronger, resulting in increased viscosity [117]. The above results clearly demonstrate that an appropriate selection of the production parameters allows for wide control of the initial and final setting times of the produced material compositions. On the other hand, the properties of geopolymer hybrids, such as density, compressive strength, and flexural strength, are significantly dependent on the content of the added amount of cement (Figure S8 in Supplementary Materials). The mechanical properties confirmed the correct choice of the materials, based on FA-0.280 + 5% cement and MK-0.350 + 5% cement, as the best mechanical properties of geopolymers (Table 4) were followed by the best mechanical properties of hybrid materials.

**Figure 3:**
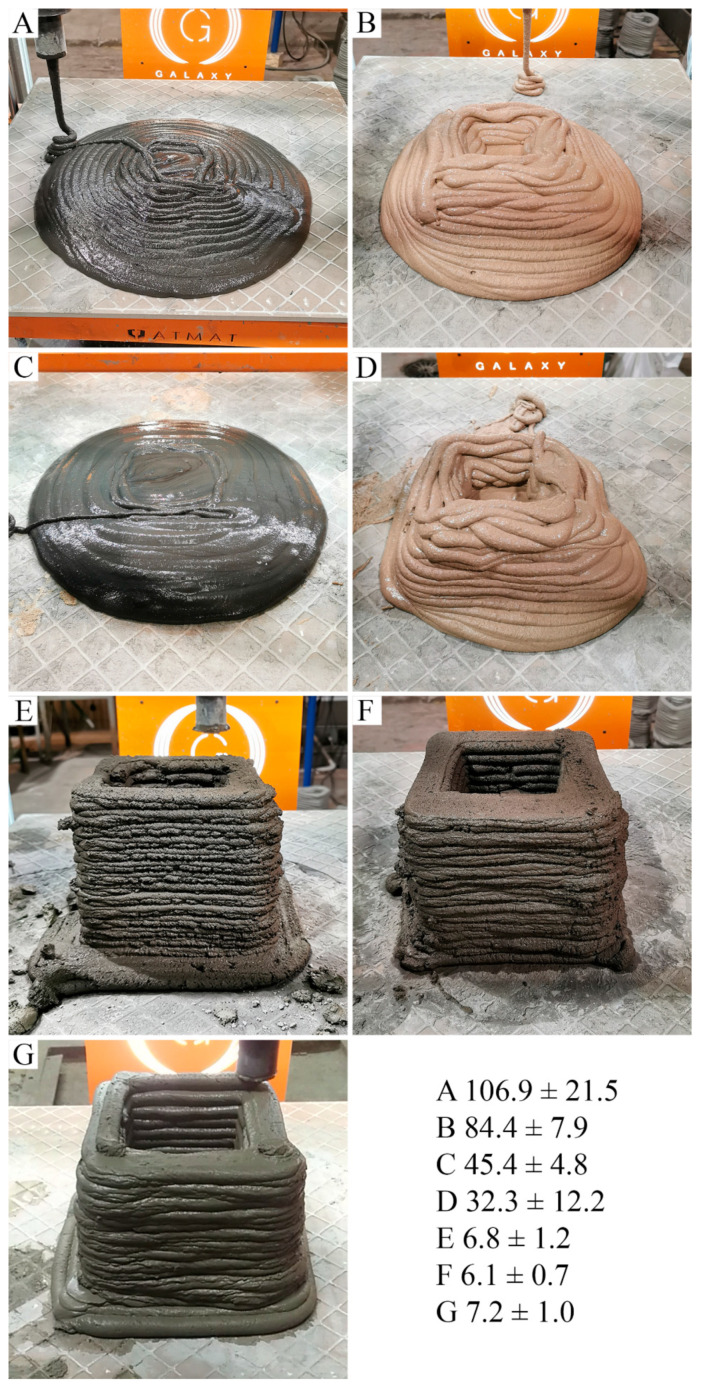
Details produced with the 3D printing method from materials with different compositions: (**A**) geopolymer based on fly ash FA-0.280; (**B**) geopolymer based on metakaolin MK-0.350, (**C**) 95% geopolymer based on fly ash FA-0.280 and 5% cement, (**D**) 95% geopolymer based on metakaolin MK-0.350 and 5% cement, (**E**) 5% geopolymer based on fly ash FA-0.280 and 95% cement, (**F**) 5% geopolymer based on metakaolin MK-0.350 and 95% cement, (**G**) cement. The values denote sample buildability (%).

A GALAXY printer (ATMAT company) was used for the printing of (1) geopolymers based on FA-0.280 and MK-0.350, (2) hybrid materials based on FA-0.280 and MK-0.350 with the addition of 5% cement, (3) hybrid materials based on cement with the addition of 5% FA-0.280 and MK-0.350, and (4) cement. Tests based on materials with various compositions indicated that the consistency of all mixtures was suitable for transport through the printer elements, ensured continuous feeding through a nozzle in the 3D printing process, and allowed the mixture to form individual layers of printed detail. However, not all materials were suitable for printing standard details. Geopolymers or hybrids based on a geopolymer matrix with the addition of 5% cement did not allow for obtaining the set dimensional parameters of the details, due to the plasticity of mortar and/or because setting time was inappropriate, and the walls of the detail were unstable. This effect was independent of the type of raw material used (FA and MK) (Figure 3A–D). Traces of residues after applying individual layers of the printed detail were seen even if the walls spilled. The materials behaved similarly to a non-Newtonian fluid. It was also found that the mixtures based on metakaolin (Figure 3B,D) had a lower spread than the tested counterpart compositions based on fly ash (Figure 3A,C). The results were confirmed by the lower values of buildability (shape stability) of the samples. This means that the ability of wet mortar to resist deformation during the layer-by-layer fabrication process was low [118]. Based on the obtained results of the setting time (Supplementary Materials Tables S5 and S7), it can be concluded that the use of this type of mixture will be effective in 3D printing technology after introducing additional modifications to the device, allowing it to heat the applied layer by volume or locally. Without additional treatments, this type of material can also be successfully used in applications aimed at the free and accurate filling of an empty or scaffold-reinforced mold. On the other hand, the use of hybrids based on cement with a 5% addition of geopolymer, based on both FA and MK, allowed for precise detail printing (Figure 3E,F). The obtained results from the visual evaluation in terms of maintaining the geometry of the shape and the quality of the printout were significantly better, compared to the results obtained for the sample printed from cement (Figure 3G). Similarly, the buildability parameters of the samples were much better. These results required that the first layer of concrete should have enough yield strength to sustain the weight of itself and the subsequent higher layers. The printed layers must be self-supporting and free of discontinuity flaws caused by insufficient cohesion or lack of continuity of material feeding. The printed layers of materials must be stacked stably to build a solid object (buildability). Furthermore, the extrudability is related to the workability of the mortar mixes. Therefore, the fresh mortar mixes dedicated to the printing process must display especially high flowability and workability during the pumping stage, whereas the requirements are just the opposite after deposition [119].

## 4. Conclusions

We present herein the development of concrete-geopolymer hybrids that are suitable for 3DCP methods and dedicated to using environmentally friendly building materials. The aim was to classify raw materials and geopolymers, as well as the design of protocols for the production of a wide range of hybrid materials with different physicochemical properties during printing but ultimately retaining the best mechanical properties as the target, including compressive strength. The compressive strength of geopolymer binders and hybrid materials were dependent on many variables in our studies, including (but not limited to): (1) the mineralogical and chemical composition of the aluminosilicate resources of the raw materials, including the Si:Al ratio, Ca, accessory minerals, dissolved organic carbon, water content, and pH; (2) the structural properties of the raw materials, with an emphasis on morphology, particle size distribution, true density, and specific surface area; (3) the physical properties of the raw materials, e.g., thermal behavior and radioactivity level. CEM I 42.5R (particle size 5–30 µm, the specific density 3.1 g cm^−3^), fly ash (spherical particles, 90% of them being < 30 µm, density 2.3 g cm^−3^), and metakaolin (irregular flake-shaped particles, 90% of them being > 30 µm, 2.6 g cm^−3^) were used as the raw materials.

The variables that should be selected to promote the geopolymerization process, i.e., greater surface area, the volume of pores, and content of silica and alumina, were found in MK, while a higher pH value, the Si:Al ratio, calcium content, and water absorption were observed in FA. In contrast, the properties reducing the geopolymerization efficiency, i.e., higher loss on ignition and the presence of dissolved organic carbon, confirmed by the thermal instability, were higher in FA. Diverse properties were not a limiting factor in the geopolymerization process. The chemical arrangement of FA-originated geopolymers (the increased Si-O-Si bonds with residual silica as reinforcement) provided better mechanical properties (compressive strength, flexural strength, and abrasion resistance) after 28 days of curing.

Similarly, many factors during the preparation process affected the properties of geopolymers and hybrid materials, which in turn determined the 3D printing process in our studies: (1) the proportion of raw materials, the liquid-to-solid ratio, the water to binder ratio; (2) the duration of mixing time and the temperature of the mixture; (3) rheology modifiers, accelerators or retarders of the setting time; (4) curing time. Therefore, changes in the liquid-to-solid ratio from 0.245 to 0.350 for FA and from 0.350 to 0.400 for MK extended the setting time by 40% and 25%, respectively. This resulted, at least, from the capillary effects (the adhesive and cohesive forces interacting between the H_2_O and the internal surface of pores) being higher in FA particles (higher Ca content, smaller particle size, the particles’ total pore volume, average pore diameter, allowing them to keep the H_2_O molecules more closely bonded; it is only when the temperature rises that adsorbed water can be removed into the atmosphere to a greater extent) than MK particles (the water is able to penetrate easily through the bigger particles and pores of the MK; a higher water quantity can be removed to the atmosphere at room temperature). Paste temperature, when increased from room temperature to a temperature of 75 °C, reduced the setting time by even one order of magnitude, while the prolongation of its mixing from 15 min to 30 min shortened the setting time twice as much. Along with an increased amount of L/S, the compressive strength decreased by ~43% and 64% after the first day of curing, for FA and MK, respectively. In the following days of curing, the effect was counteracted in the wide range of the L/S ratio for FA-originated geopolymers, but not MK-originated geopolymers. With the introduction of cement to geopolymer mortar in the range of 5–30%, the setting time of the hybrid material was shortened, while the content of cement in the range of 95% and 100% prolonged the setting time. Many other variables, as optimized earlier, can also modify the physico-mechanical properties of the final product, e.g.: (1) the type and formulation of the alkaline activator and the content of alkaline ions in the activator; the fraction of silicate to hydroxide compounds in the activator [26,120]; (2) the formulation of aggregates [75,76,77,78,80]; and (3) specific modifications, including reinforcing additives [59,121], agents controlling the geopolymer macrostructure [58,59], and curing conditions.

The results of the 3D printing of cement-geopolymer hybrids were presented for the first time. Geopolymers or hybrids based on a geopolymer matrix with the addition of 5% cement resulted in a high setting time, and the final materials behaved similarly to a non-Newtonian fluid. The use of this type of mixture is recommended in 3D printing technology after heating the printed elements. Without additional treatments, this type of material can be successfully used to fill the molds. In contrast, hybrid materials based on cement with a 5% addition of geopolymer, based on both FA and MK, enabled precise detail printing. Future work should consist of the optimization of printing. Particularly when the geopolymer binder is 3D-printed, the number of effective factors on the product strength is expanded by: (1) the printing method, size and geometry of printing nozzle, the number of nozzles; (2) the printing parameters, which include the resolution of layers, mainly Z-layer thickness, degree, and shape of the extrusion (circular, ovular or rectangular), linear rates of extrusion, the orientation of manufacture (vertical or horizontal), retraction; (3) mass sensitivity to mechanical and physical factors.

Altogether, our findings demonstrate a great ability to achieve the classification of geopolymers for different 3D-printing methods and imply the significance of different factors in the compressive strength of the final product. However, mixture design and the appropriate selection of ingredients in laboratory conditions are costly and time-consuming; therefore, to achieve the desired mechanical properties and to support the usage of FA in the building industry, data can be applied in future research using data-driven methods involving artificial intelligence and machine learning methods, as well as reliable, precise, and accurate mathematical equations [56,122,123].

## Figures and Tables

**Table 1:** Type of geopolymer samples.

Sample	Composition, wt. %			Liquid/Solid Ratio
	FA: Sand 1:1	MK: Sand 1:1	10M NaOH: Water Glass 1:2.5	
FA-0.245	80.32	-	19.68	0.25
FA-0.280	78.12	-	21.88	0.28
FA-0.350	74.07	-	25.93	0.35
MK-0.350	-	74.07	25.93	0.35
MK-0.375	-	72.73	27.27	0.38
MK-0.400	-	71.43	28.57	0.40

**Table 2:** Textural characteristics of fly ash and metakaolin, BET—specific surface area analysis method; BJH—pore size and volume analysis method.

Parameter	FA	MK
Single-Point BET (m^2^ g^−1^)	10.431	12.999
Multi-Point BET (m^2^ g^−1^)	12.760	15.315
Surface Area BET (m^2^ g^−1^)	14.616	21.415
Total pore volume BJH (cm^3^ g^−1^)	0.026	0.140
Pore volume BJH (cm^3^ g^−1^)	0.027	0.142
Average pore diameter BJH (nm)	2.134	2.975

**Table 3:** Fly ash and metakaolin chemical compositions (X-ray fluorescence), loss on ignition (LOI), and mineral phases as calculated from the XRD data (Figure S3 in the Supplementary Materials) with the Rietveld method results.

Main Minerals, %			Accessory Minerals, ppm			Mineral Phases, %		
	FA	MK		FA	MK		FA	MK
SiO_2_	48.220	52.430	BaO	800.0	99.7	Quartz	42.0	9.4
TiO_2_	1.110	0.310	SrO	600.0	103.5	Mullite	52.5	5.8
Al_2_O_3_	26.130	42.750	Zn	199.7	37.1	Hematite	2.6	-
Fe_2_O_3_	7.010	1.200	Pb	146.4	151.7	Magnetite	1.0	-
MnO	0.090	0.012	V	274.2	34.6	Anhydrite	1.2	-
MgO	1.720	0.175	Cr	171.4	-	Rutile	0.7	-
CaO	5.120	0.490	Cu	129.8	15.3	Illite-2M1	-	43.4
Na_2_O	1.615	0.000	Ni	109.6	-	Kaolinite-1A	-	41.4
K_2_O	3.480	1.300	Rb	184.0	156.6			
P_2_O_5_	0.700	0.440	Ga	31.6	57.6			
SO_3_	1.110	0.030	Zr	209.0	84.0			
Cl	0.090	0.060	Te	40.1	22.5			
LOI	3.284	0.722	As	-	20.4			
			Sb	20.6	-			
			Sn	45.2	37.0			
			Y	49.1	17.7			

**Table 4:** Mechanical properties of geopolymers produced from fly ash and metakaolin after 1 and 28 days of curing, represented by compressive strength (MPa) after 1 day and 28 days of curing, as well as flexural strength (MPa) and abrasion resistance (cm^3^ 50 cm^−2^ and %) after 28 days of curing. The representative photos of the samples after tests are presented in Figure S6 in the Supplementary Materials.

Sample	Compressive Strength		Flexural Strength	Abrasion Resistance	
	1 Day	28 Days	28 Days	28 Days	28 Days
FA-0.245	44.73 ± 8.05	39.55 ± 3.29	7.58 ± 0.54	26.27 ± 9.67	7.20 ± 2.64
FA-0.280	41.71 ± 11.27	47.47 ± 1.12	9.38 ± 0.36	36.07 ± 2.21	9.81 ± 0.67
FA-0.350	25.45 ± 2.75	40.43 ± 7.20	5.68 ± 0.33	14.22 ± 1.72	3.82 ± 0.46
MK-0.350	68.34 ± 2.64	53.24 ± 3.78	6.25 ± 0.56	12.40 ± 0.04	3.34 ± 0.04
MK-0.375	61.40 ± 7.90	34.65 ± 4.69	4.50 ± 0.02	30.17 ± 13.24	8.50 ± 3.96
MK-0.400	24.62 ± 0.52	34.23 ± 3.26	4.29 ± 0.67	18.24 ± 4.29	4.70 ± 1.14

## Data Availability

Not applicable.

## Supplementary Materials

The following are available online at https://www.mdpi.com/article/10.3390/ma14226874/s1, Figure S1. SEM micrographs of fly ash and metakaolin at different magnifications (100×, 200×, 1000×). A,C,E—fly ash morphology, B,D,F—metakaolin morphology. Figure S2. The nitrogen adsorption–desorption isotherms of fly ash and metakaolin. Adsorption isotherms exhibit shapes that depend on the intensity of the adsorbate-adsorbent interaction and on the pore size. According to the IUPAC classification, N2 sorption isotherms can be classified as type IV, which indicates the presence of mesopores. The hysteresis loops are of type H3 for slit-like interparticle pores. Figure S3. Thermal decomposition of the fly ash and metakaolin during heating from ambient temperature to 1000 °C: A—thermogravimetry (TG, solid lines) and DTG (derivative thermogravimetry, dashed lines) curves; B—differential thermal analysis (DTA) curves; C–H—curves of evolved gas analysis (Quadrupole Mass Spectrometry) for C—*m/z* 12, H_2_O—*m/z* 17 and 18, CO—*m/z* 28, O_2_—*m/z* 32, CO_2_—*m/z* 44, respectively. Figure S4. XRD patterns of fly ash and metakaolin. Figure S5. Complete FTIR spectra of raw materials, i.e., fly ash and metakaolin (A), as well as geopolymers produced from fly ash (B) and metakaolin (C), mixed with sand and NaOH:water glass in ratio 0.245, 0.280 and 0.350 for FA, and 0.350, 0.375, and 0.400 for MK. The spectra correspond to Figure 2. Figure S6. Photographs of representative geopolymer samples after an analysis of compressive strength after 1 day and 28 days of curing, as well as flexural strength and abrasion resistance after 28 days of curing. Photographs of fly ash- and metakaolin-based geopolymers with the same liquid-to-solid ratio of 0.35 were compared. Figure S7. Initial and final setting time (min) of hybrid samples based on fly ash FA-0.280 and the different contents of cement. The duration of the ingredient-mixing before the test was set to 15 min and the experiment was carried out at room temperature, according to the EN 196-2: 2005 + A1: 2008 standard with 600 cm^−3^ of mortar. Standard error did not exceed 10%. Figure S8. Density (A), compressive strength (B), and flexural strength (C) of printed hybrid samples based on fly ash (FA-0.280), depending on the content of the added cement and carried out at room temperature. The duration of ingredients mixing before the test was set to 15 min. The tests were carried out according to the EN 196-2: 2005 + A1: 2008 standard with 600 cm^−3^ of mortar. Standard error did not exceed 10%. Table S1. The particle size distribution width (μm) of the fly ash and metakaolin. The distribution width corresponded to the data presented on Figure 1C in the main text. The D_50_, the median, has been defined as the diameter where half of the population lies below this value. Similarly, 90 percent of the distribution lies below the D_90_, and 10 percent of the population lies below the D_10_. Table S2. Composite of the water leachates tested for fly ash and metakaolin presented in mg L^−1^. Table S3. Natural radioactivity testing of raw materials presented in Bq kg^−1^. Table S4. Main FTIR bands of raw materials i.e., fly ash and metakaolin (A) as well as geopolymers produced from fly ash (B) and metakaolin (C) mixed with sand and NaOH: water glass in ratio 0.245, 0.280 and 0.350 for FA, and 0.350, 0.375, and 0.400 for MK. The bands are related to Figure 2 and Supplementary Materials Figure S5. Table S5. Initial and final setting time (min) of geopolymer samples based on fly ash and metakaolin tested at 75 °C. The duration of ingredient-mixing before the test was 15 and 30 min. The tests were carried out according to the EN 196-2: 2005 + A1: 2008 standard with 600 cm^−3^ of mortar. Standard error did not exceed 10%. Table S6. Consistency of fresh geopolymer mortars determined by the flow table method (mm) and the Novikov cone method (mm). The duration of ingredient-mixing was 15 min, room temperature. The flow table method was carried out according to the EN 1015-3. 1500 cm^−3^ of mortar was taken into the mold, the measurement was taken as the average mortar spreading (mean diameter) measured in two directions perpendicular to each other (diameter 1 and diameter 2). According to the EN 1015-3-6 standard, the mortar consistency is defined as: dense-plastic with the value < 140, plastic for the values in the range of 140–200, and liquid with the value > 200. Novikov’s cone method was performed according to the PN-85/B-04500 standard, by determining the resistance of the mortar to a free-immersion cone with a mass of 300 g in about 1 dm^3^ of mass. The measurement value corresponds to the depth of the cone immersion into the geopolymer mortar and is read in centimeters along the cone. Standard error did not exceed 10%. Table S7 Initial and final setting time (min) of hybrid samples based on fly ash FA-0.280 and metakaolin MK-0.350 with 5% addition of cement, carried out at room temperature and 75 °C. The duration of ingredient-mixing before the test was set to 15 min and the experiment was carried out at room temperature, according to the EN 196-2: 2005 + A1: 2008 standard, with 600 cm^−3^ of mortar. Standard error did not exceed 10%.
