# Supplementary material for: Hybrid Materials Based on Fly Ash, Metakaolin, and Cement for 3D Printing
Source: Materials (Basel). 2021 Nov 15;14(22):6874. doi: 10.3390/ma14226874 (PMC8618050; doi:10.3390/ma14226874)
Supplement: Supplementary file 1 [file materials-14-06874-s001.zip › materials-1427480-supplementary.pdf]

Article

Hybrid materials based on fly ash, metakaolin, and cement for 3D printing

Joanna Marczyk[1], Celina Ziejewska[1], Szymon Gądek[1], Kinga Korniejenko[1], Michał Łach[1], Mateusz Góra[1,2], Izabela Kurek[1], Neslihan Doğan-Sağlamtimur[3], Marek Hebda[1*], Magdalena Szechyńska-Hebda[4*]

[1] Faculty of Material Engineering and Physics, Cracow University of Technology, Warszawska 24, 31-155 Kraków, Poland
[2] ATMAT Sp. z o.o., Siwka 17, 31-588 Kraków, Poland
[3] Department of Environmental Engineering, Faculty of Engineering. Nigde Omer Halisdemir University, Nigde, Turkey
[4] Plant Breeding and Acclimatization Institute - National Research Institute, Radzików, 05-870 Błonie, Poland
* Correspondence: mhebda@pk.edu.pl, szechynska@wp.pl, tel.: +48 126283423

Supplementary Materials

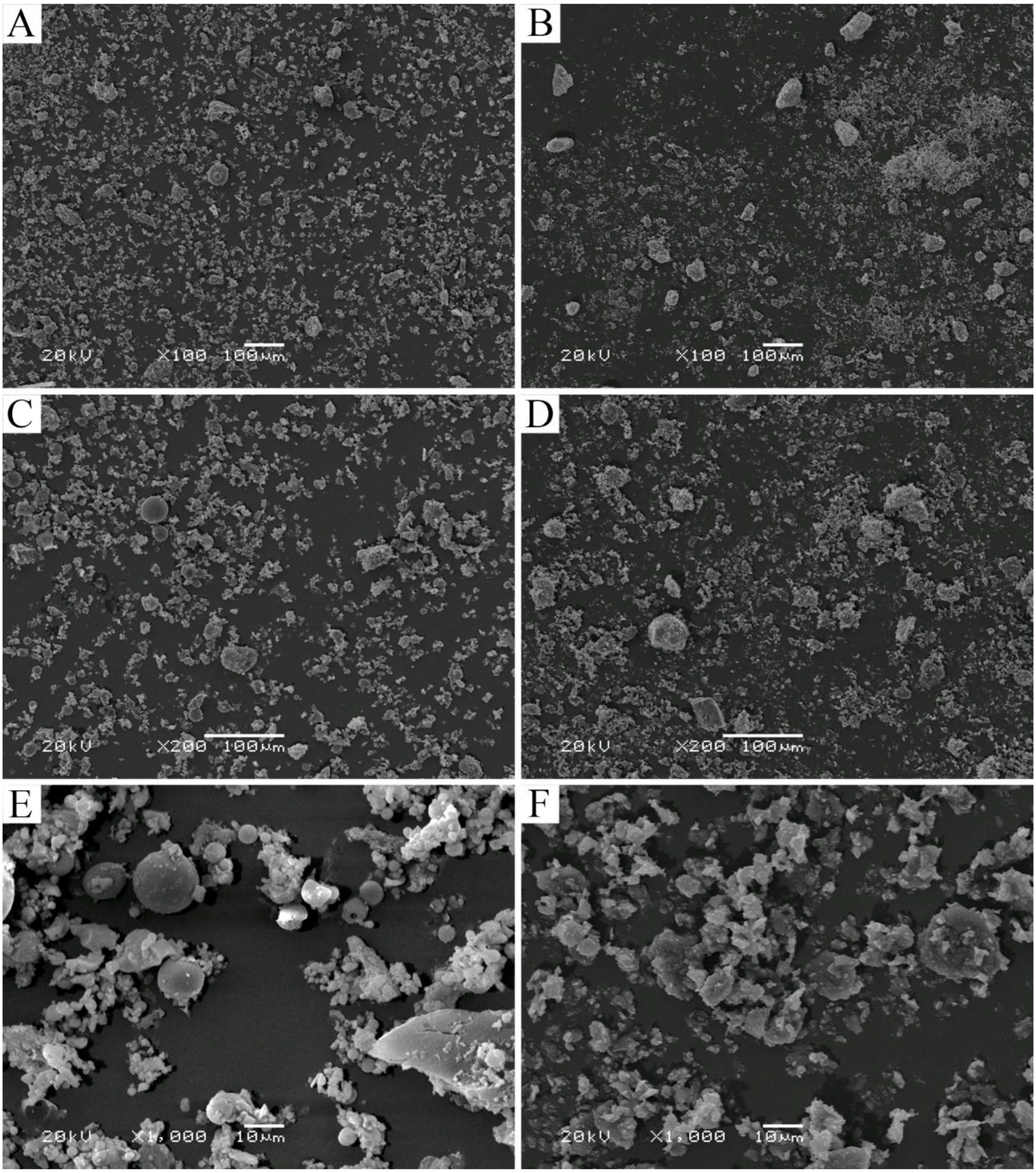

Figure S1. SEM micrographs of fly ash and metakaolin at different magnifications (100x, 200x, 1000x). A, C, E - fly ash morphology, B, D, F - metakaolin morphology.

Table S1. The particle size distribution width (µm) of the fly ash and metakaolin. The distribution width corresponded to data presented on Figure 1C in the main text. The D_{50}, the median, has been defined as the diameter where half of the population lies below this value. Similarly, 90 percent of the distribution lies below the D_{90}, and 10 percent of the population lies below the D_{10}.

**Distribution width**	**FA**	**MK**
D5	1.3	0.5
D10	2.8	0.8
D25	18.9	1.5
D50 (median)	22.3	18
D75	25.4	25.4
D90	28.2	30.8
D95	29.6	33.9
D99	32.5	39.2

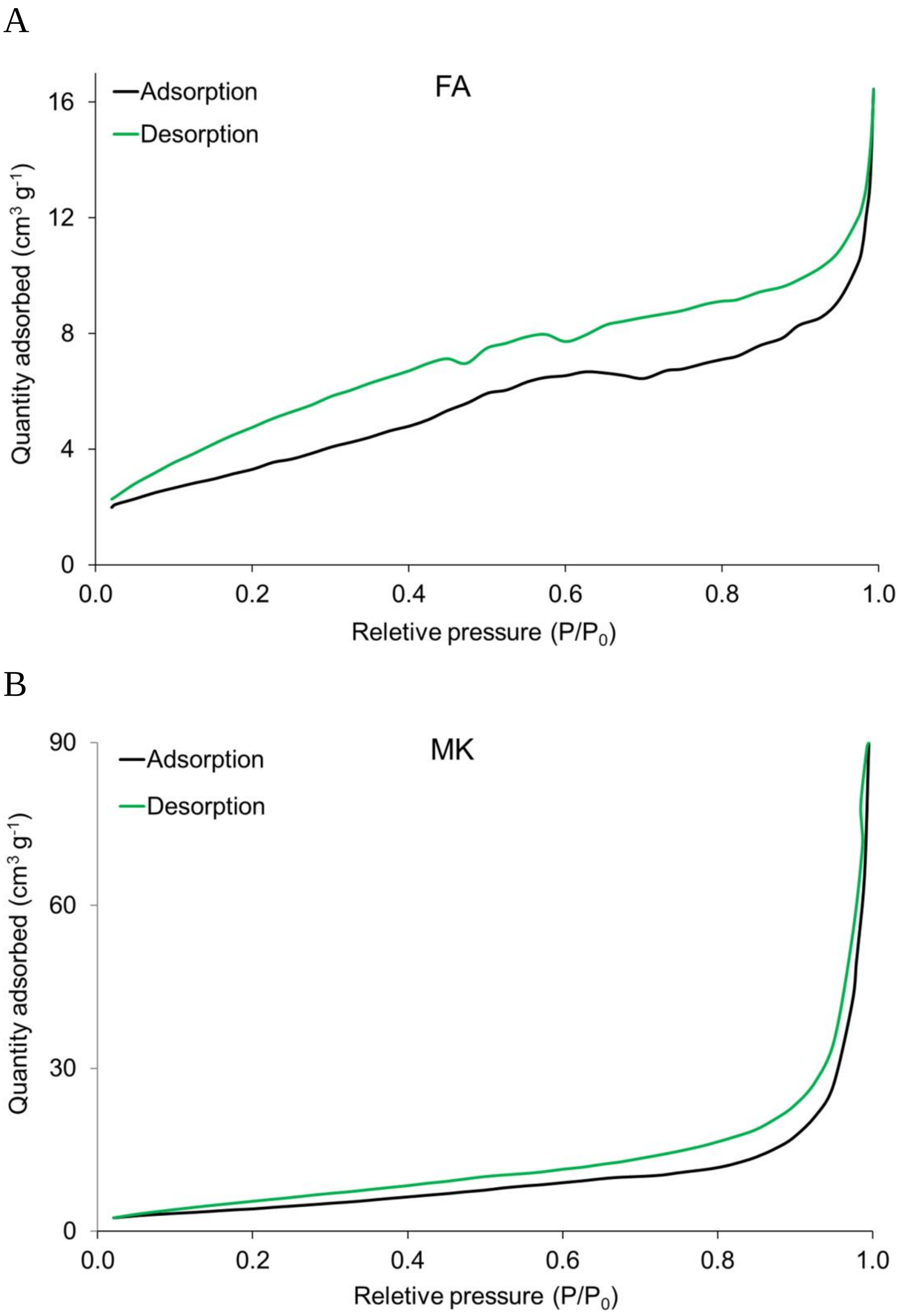

Figure S2. The nitrogen adsorption–desorption isotherms of fly ash and metakaolin. Adsorption isotherms exhibit shapes that depend on the intensity of the adsorbate-adsorbent interaction and on the pore size. According to the IUPAC classification, N2 sorption isotherms can be classified as type IV, which indicates the presence of mesopores. The hysteresis loops are of type H3 for slit-like interparticle pores.

Table S2. Composite of the water leachates tested for fly ash and metakaolin presented in mg l^{-1}.

Component	FA	MK
Sb	< 0.01	< 0.005
As	< 0.01	0.018
Ba	0.66	0.051
Cr	0.12	< 0.005
Cr(VI)	0.12	< 0.01
Zn	< 0.03	< 0.03
Cd	< 0.001	< 0.001
Cu	< 0.005	< 0.005
Mo	0.29	0.0068
Ni	< 0.005	< 0.005
Pb	< 0.005	< 0.005
Hg	< 0.001	< 0.001
Se	0.030	< 0.005
chlorides	20	< 5
fluorides	2.4	0.78
sulphates	414	44
DOC (dissolved organic carbon)	3.6	2.8
total dissolved substances	1390	100

Table S3. Natural radioactivity testing of raw materials presented in Bq kg^{-1}.

Determinable parameter	FA	MK
Potassium activity concentration (^{40}K)	814.06 ± 73.27	353.77 ± 31.84
Radium activity concentration (^{226}Ra)	194.33 ± 23.32	75.83 ± 9.10
Thorium activity concentration (^{228}Th)	100.95 ± 28.27	128.61 ± 36.02

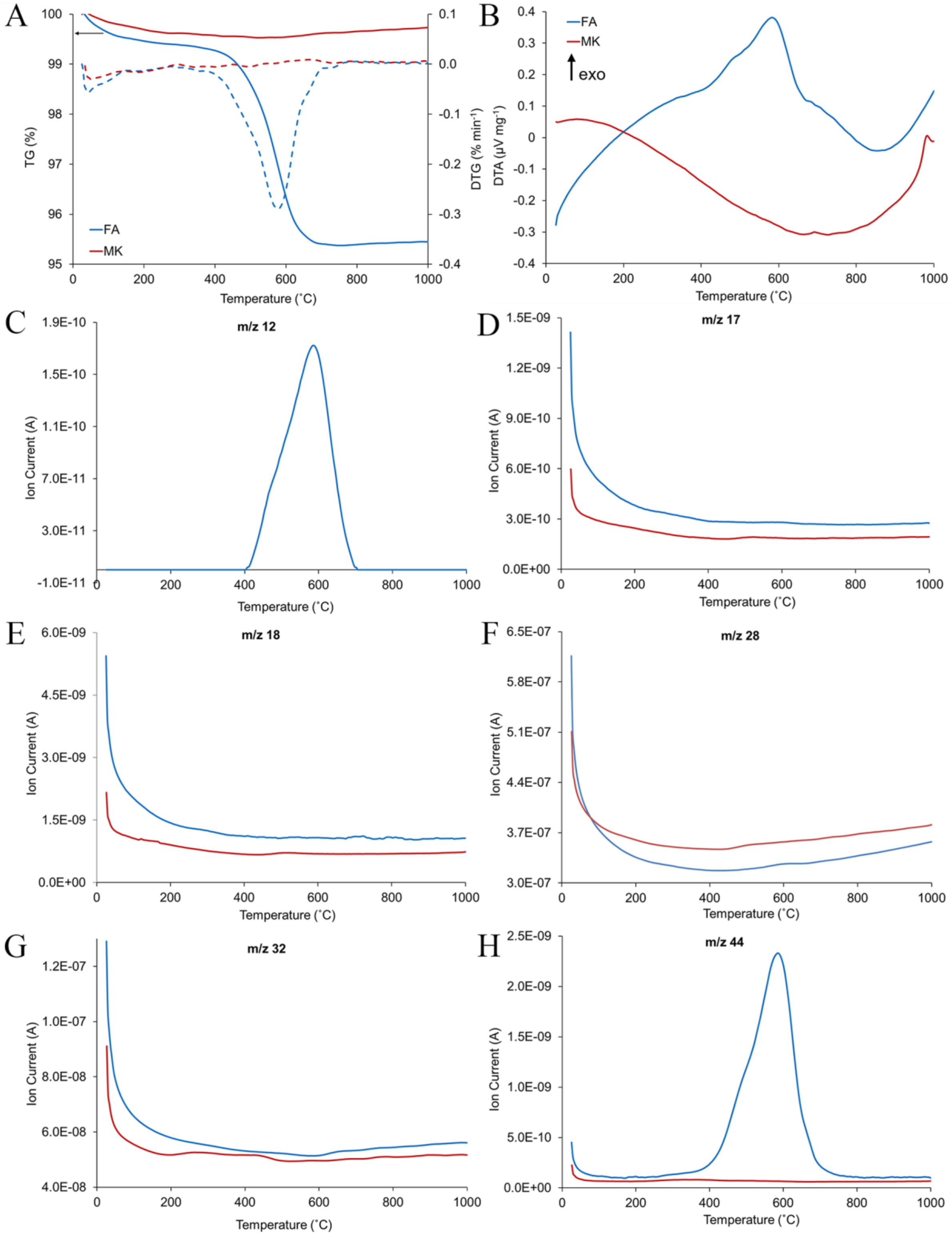

Figure S3. Thermal decomposition of the fly ash and metakaolin during heating from ambient temperature to 1000 °C: A – thermogravimetry (TG, solid lines) and DTG (derivative thermogravimetry, dashed lines) curves; B – differential thermal analysis (DTA) curves; C-H – curves of evolved gas analysis (Quadrupole Mass Spectrometry) for C - *m*/*z* 12, H_2O - *m*/*z* 17 and 18, CO - *m*/*z* 28, O_2 - *m*/*z* 32, CO_2 - *m*/*z* 44, respectively.

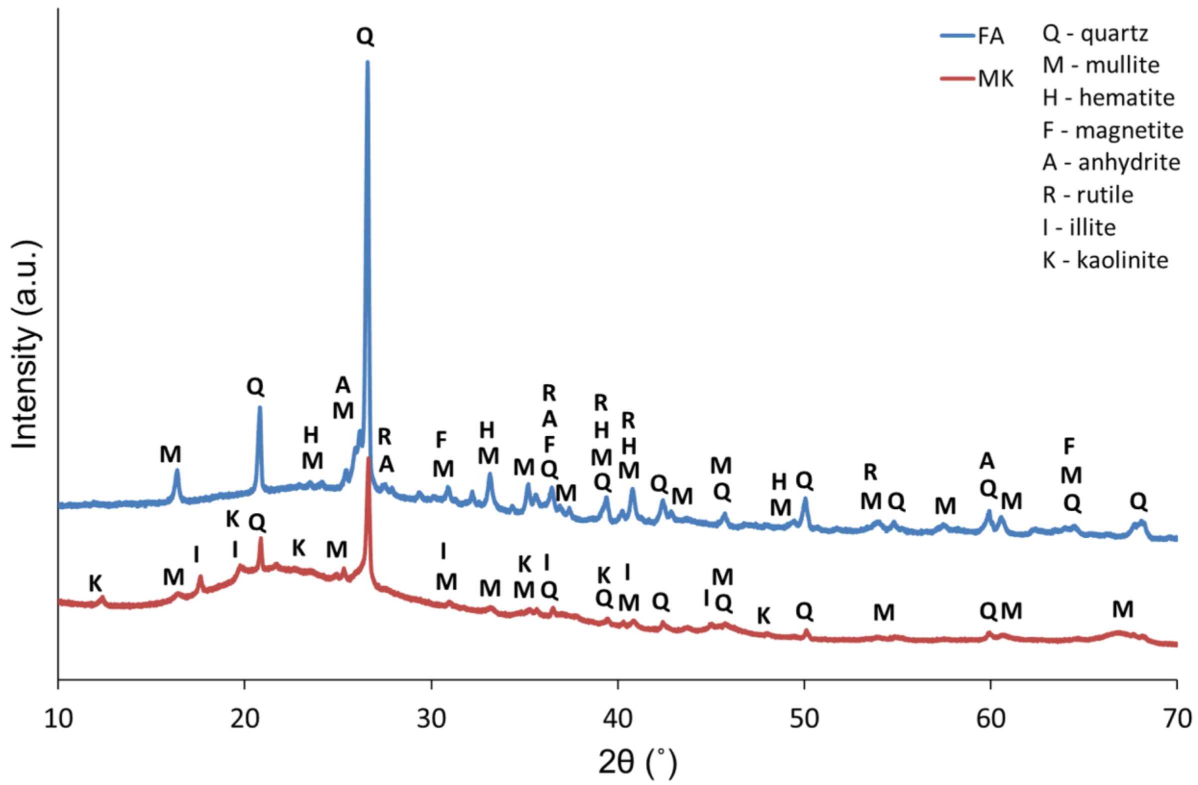

Figure S4. XRD patterns of fly ash and metakaolin.

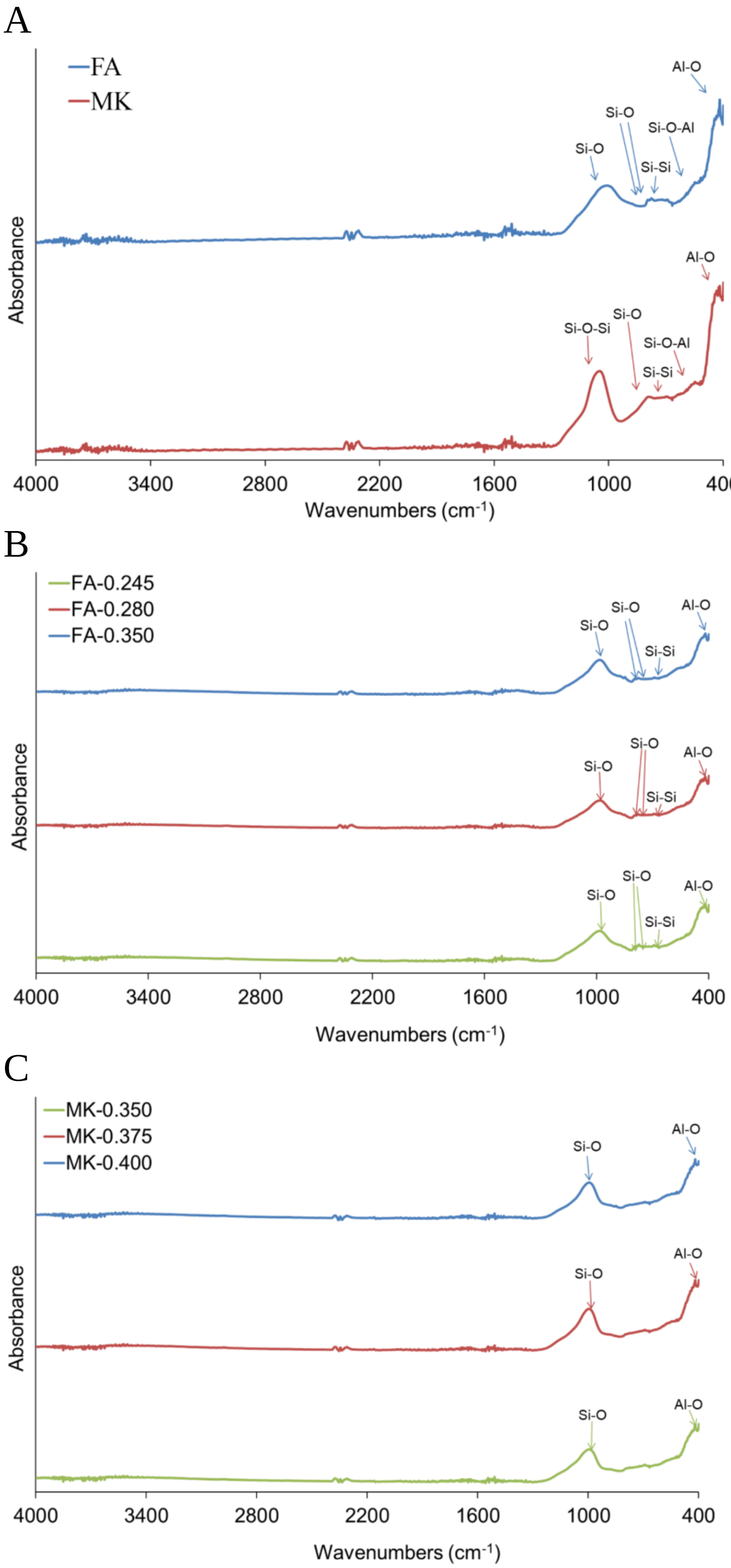

Figure S5. Complete FTIR spectra of raw materials i.e. fly ash and metakaolin (A) as well as geopolymers produced from fly ash (B) and metakaolin (C) mixed with sand and NaOH : water glass in ratio 0.245, 0.280 and 0.350 for FA, and 0.350, 0.375, and 0.400 for MK. The spectra correspond to Figure 2.

Table S4. Main FTIR bands of raw materials i.e. fly ash and metakaolin (A) as well as geopolymers produced from fly ash (B) and metakaolin (C) mixed with sand and NaOH : water glass in ratio 0.245, 0.280 and 0.350 for FA, and 0.350, 0.375, and 0.400 for MK. The bands are related to Figure 2 and Supplementary Materials Figure 5.

**Sample**	**Wavenumber**	**Vibration band**
	Raw materials	
FA	1003 cm-1	Vibration of Si-O
	793 cm-1 and 781 cm-1	Bending vibration of Si-O
	699 cm-1	Vibration of Si-Si
	547 cm-1	Vibration of Si-O-Al
	418 cm-1 and adjacent bands	Vibration of Al-O
MK	1055 cm-1	Vibration of Si-O-Si
	793 cm-1	Bending vibration of Si-O
	699 cm-1	Vibration of Si-Si
	547 cm-1	Vibration of Si-O-Al
	418 cm-1 and adjacent bands	Vibration of Al-O
	Geopolymers	
FA	989 cm-1	Vibration of Si-O
	793 cm-1 and 783 cm-1	Bending vibration of Si-O
	690 cm-1	Vibration of Si-Si
	420 cm-1	Vibration of Al-O
MK	992 cm-1	Vibration of Si-O
	420 cm-1	Vibration of Al-O

Sample	Before test	After test<br>1 day of curing	 28 days of curing
Compressive strength			
FA	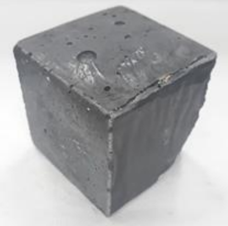	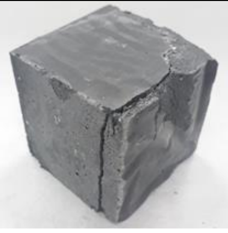	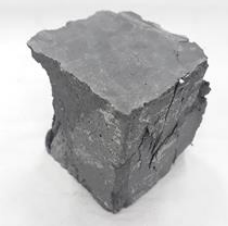
MK	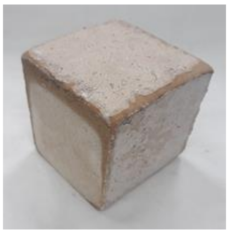	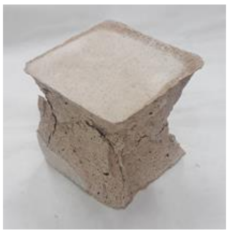	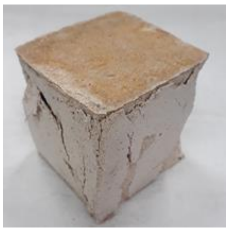
Flexural strength			
FA	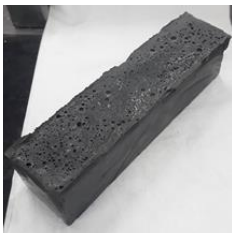		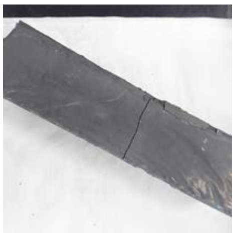
MK	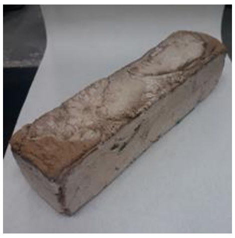		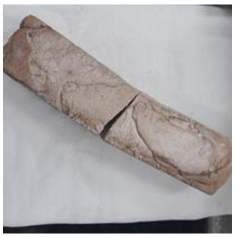
Abrasion resistance			
FA	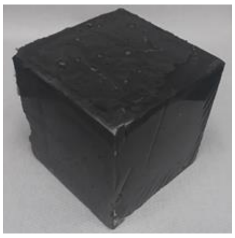		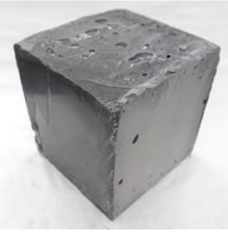
MK	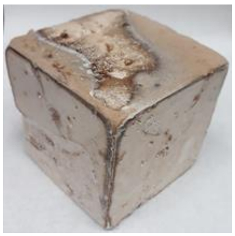		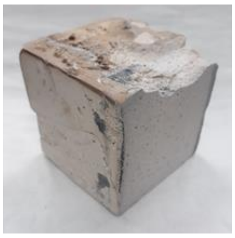

Figure S6. Photographs of representative geopolymer samples after the analysis of: compressive strength after 1 day and 28 days of curing, as well as flexural strength and abrasion resistance after 28 days of curing. Photographs of fly ash- and metakaolin-based geopolymers with the same liquid-to-solid ratio of 0.35 were compared.

Table S5. Initial and final setting time (min) of geopolymer samples based on fly ash and metakaolin tested at 75 °C. The duration of ingredients mixing before test was 15 and 30 minutes. The tests were carried out according to the EN 196-2: 2005 + A1: 2008 standard with 600 cm^{-3} of mortar. Standard error did not exceed 10%.

Sample	Initial time	Final time	Initial time	Final time	Initial time	Final time
	15 min + RT		15 min + 75 °C		30 min + 75 °C	
FA-0.245			27	34		
FA-0.280	405	630	32	40	14	18
FA-0.350			40	46		
MK-0.350			28	33		
MK-0.375	323	522	35	38	29	34
MK-0.400			37	39		

Table S6. Consistency of fresh geopolymer mortars determined by flow table method (mm) and the Novikow cone method (mm). The duration of ingredients mixing was 15 minutes, room temperature. The flow table method was carried out according to the EN 1015-3. 1500 cm^{-3} of mortar was taken into the mould, the measurement was taken as the average mortar spreading (mean diameter) measured in two directions perpendicular to each other (diameter 1 and diameter 2). According to the EN 1015-3-6 standard, the mortar consistency is defined as: dense-plasticic with the value < 140, plasticic for the values in the range of 140-200, and liquid with the value > 200. The Novikov`s cone method was performer according to the PN-85/B-04500 standard, by determining the resistance of the mortar to a freely immersion cone with a mass of 300 g in about 1 dm^3 of mass. The measurement value corresponds to the depth of the cone immersion into the geopolymer mortar and is read in centimeters along the cone. Standard error did not exceed 10%.

Sample	Flow table method			Novikov`s cone method
	Diameter 1	Diameter 2	Mean	
FA-0.245	100	105	103	100
FA-0.280	135	140	138	800
FA-0.350	255	260	258	1100
MK-0.350	105	110	108	350
MK-0.375	150	145	148	250
MK-0.400	180	180	180	100

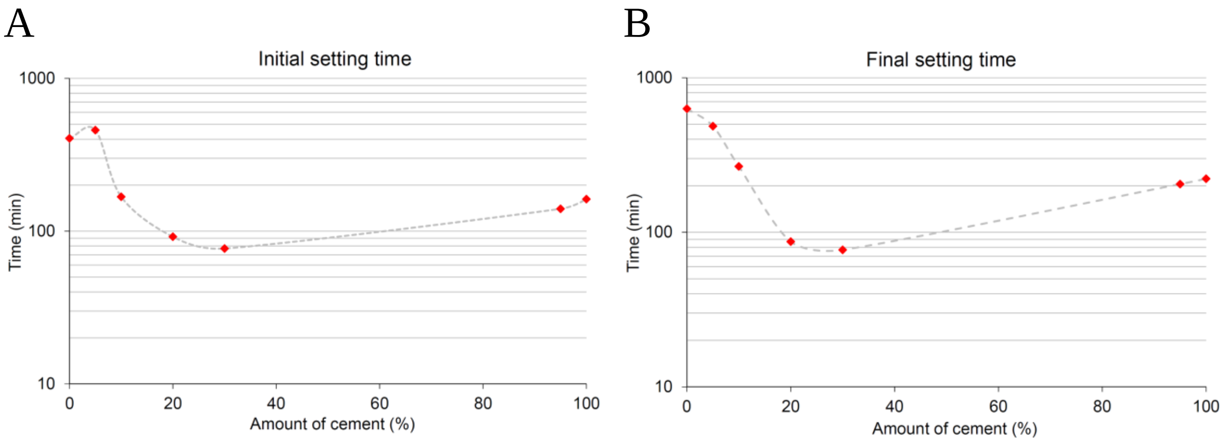

Figure S7. Initial and final setting time (min) of hybrid samples based on fly ash FA-0.280 and different content of cement. The duration of ingredients mixing before test was set to 15 minutes and experiment was carried out at room temperature according to the EN 196-2: 2005 + A1: 2008 standard with 600 cm^{-3} of mortar. Standard error did not exceed 10%.

Table S7 Initial and final setting time (min) of hybrid samples based on fly ash FA-0.280 and metakaolin MK-0.350 with 5% addition of cement. carried out at room temperature and 75 °C. The duration of ingredients mixing before test was set to 15 minutes and experiment was carried out at room temperature according to the EN 196-2: 2005 + A1: 2008 standard with 600 cm^{-3} of mortar. Standard error did not exceed 10%.

Sample	Initial time	Final time	Initial time	Final time
	RT		75 °C	
FA-0.280 + 5% cement	459	486	49	55
MK-0.350 + 5% cement	366	403	31	44

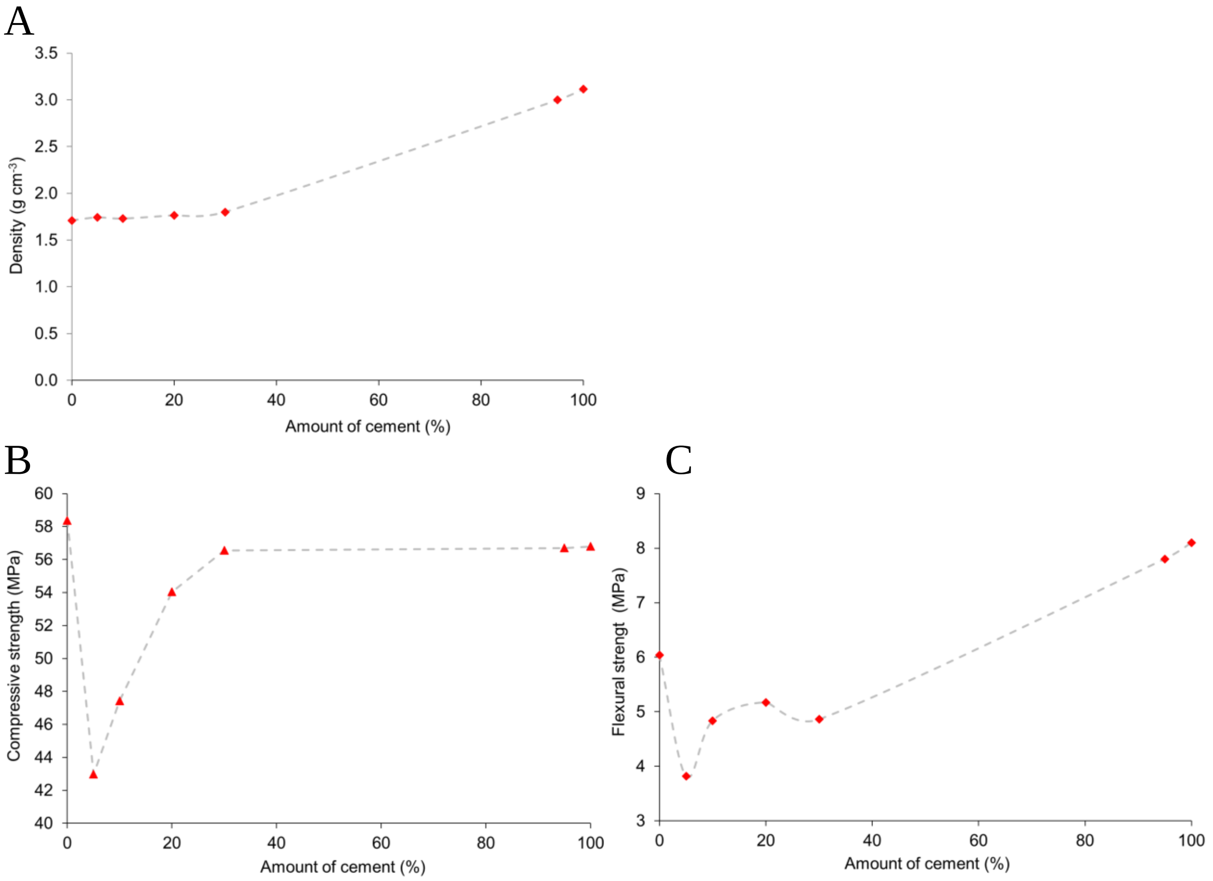

Figure S8. Density (A), compressive strength (B), and flexural strength (C) of printed hybrid samples based on fly ash (FA-0.280) depending on the content of the added cement and carried out at room temperature. The duration of ingredients mixing before test was set to 15 minutes. The tests were carried out according to the EN 196-2: 2005 + A1: 2008 standard with 600 cm^{-3} of mortar. Standard error did not exceed 10%.
